# A correlation study of biological activity and molecular docking of Asp and Glu linked bis-hydrazones of quinazolinones†

**DOI:** 10.1039/c8ra00531a

**Published:** 2018-03-16

**Authors:** H. K. Kumara, R. Suhas, D. M. Suyoga Vardhan, M. Shobha, D. Channe Gowda

**Affiliations:** Department of Studies in Chemistry, University of Mysore Manasagangotri Mysuru – 570 006 Karnataka India dchannegowda@yahoo.co.in +91 821 2419664

## Abstract

The present investigation involves the synthesis and spectroscopic and biological activity studies of the bis-hydrazones of quinazolinones derived from aspartic acid and glutamic acid. The antioxidant activities of the compounds were evaluated using DPPH, DMPD and ABTS radical scavenging assays whose results revealed that the IC_50_ of compounds 6, 7, 11, 12, 20, 21, 25 and 26 was lower than those of the standard references. The anti-inflammatory activity was evaluated with a haemolysis assay using a human blood erythrocytes suspension and the results demonstrated that compounds 8, 9, 13, 14, 22, 23, 27 and 28 were excellent anti-inflammatory agents. In addition, the antibacterial and antifungal activities against various clinical pathogens of human origin revealed that compounds 7, 9, 12, 14, 21, 23, 26 and 28 possessed potent antimicrobial properties. Furthermore, to understand the correlation between biological activity and drug–receptor interaction, molecular docking was performed on the active sites of tyrosine kinase (PDB ID: 2HCK), cyclooxygenase-2 (PDB ID: 1CX2) and glucosamine-6-phosphate (GlcN-6-P) synthase (PDB ID: 2VF5) which showed good binding profiles with the targets that can potentially hold the title compounds. The correlation study revealed that compounds containing EDGs (–OH, –OCH_3_) were excellent antioxidants, compounds with EWGs (–Cl, –NO_2_) exhibited good anti-inflammatory activity and compounds bearing –OH and –NO_2_ groups were very good antimicrobials.

## Abstract 2

The present investigation involves the synthesis and spectroscopic and biological activity studies of the bis-hydrazones of quinazolinones derived from aspartic acid and glutamic acid.

## Graphical abstract

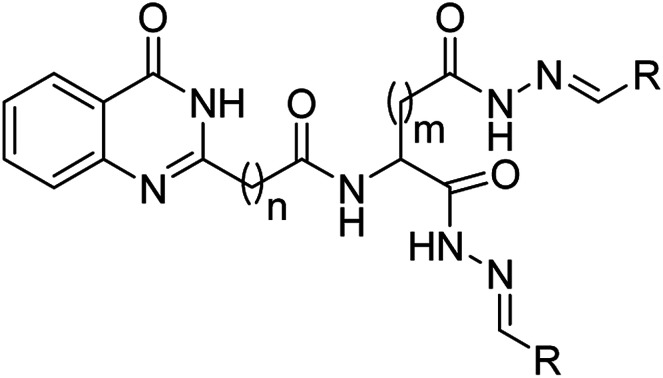

## 1. Introduction

Computational biology and bioinformatics play a major role in designing drug molecules and have the potential to speed up the drug discovery process. Molecular docking of the drug molecule with the receptor gives important information about drug–receptor interactions and is commonly used to find out the binding orientation of drug candidates to their protein targets in order to predict the affinity and activity.^1^ Medicinal chemistry is a specialized science that has evolved to encompass a broad range of disciplines concerned with the identification, synthesis and development of drug-like compounds for therapeutic use. It needs a wide range of expertise, developed through years of training, dedication and learning from best practice in order to produce drugs that are good enough to enter clinical trials with patients.^2^

**Fig. 1:**
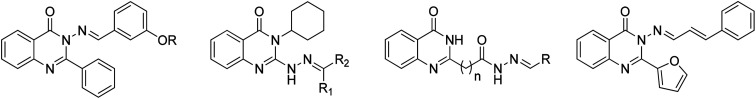
The structures of the biologically active quinazolinone-hydrazones.

Quinazolinones and their derivatives are building blocks of approximately 150 naturally occurring alkaloids isolated from plants, animals and microorganisms. The quinazolinone nucleus has diverse pharmacological activities, including antimicrobial,^3^ anti-inflammatory,^4^ antimalarial,^5^ anticonvulsant,^6^ antihypertensive,^7^ anti-diabetic,^8^ PARP inhibitory^9^ and anticancer activity.^10^ On the other hand, hydrazones are present in many bioactive heterocyclic compounds and are of wide interest because of their diverse biological and clinical applications. Moreover, hydrazones of quinazolinone derivatives exhibited enhanced biological activity, *viz.* antimicrobial,^11^ anti-inflammatory,^12^ antiviral,^13^ analgesic^14^ and anticancer activity.^15^ The structures of the biologically active quinazolinone-hydrazones are shown in Fig. 1.

Besides these, amino acids are endogenous substances which could be used for the modification of drug skeletons to promote the absorption of drugs.^16^ In addition, the toxicity of drugs also could be reduced *via* the introduction of amino acids.^17^ Conjugation of amino acids/peptides with heterocycles,^18,19^ especially with a quinazolinone moiety,^20–22^ enhances the overall therapeutic properties of the molecule. Motivated by the aforementioned literature and the recent encouraging results from our research group,^23–25^ we have directed systematic efforts towards the synthesis of new bis-hydrazones of Asp and Glu linked quinazolinone derivatives followed by the study of their antimicrobial, antioxidant and anti-inflammatory activity. In addition, the results obtained were correlated with molecular docking studies in order to obtain some information about the binding efficiency and target interactions. The above are all collectively presented in this communication.

## 2. Results and discussion

### 2.1. Chemistry

**Scheme 1:**
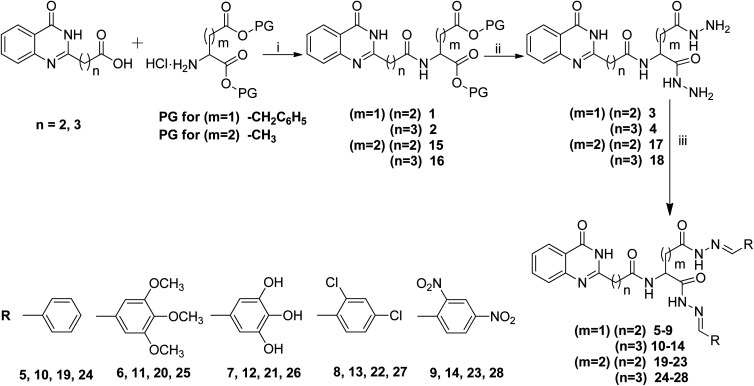
Bis-hydrazones of quinazolinone derived from anionic amino acid linkers. Reagents and conditions: (i) NMM, EDCI/HOBt, 0 °C to rt, (ii) NH_2_NH_2_·H_2_O, ethanol, reflux, 16 h, (iii) R–CHO, EtOH, reflux, 7–8 h.

The synthesis of the desired compounds was achieved according to the steps illustrated in Scheme 1. 3-(4-Oxo-3,4-dihydroquinazolin-2-yl)propanoic acid (QZN 1) and 4-(4-oxo-3,4-dihydroquinazolin-2-yl)butanoic acid (QZN 2) were synthesized by literature known methods.^26–28^ Conjugation of QZN 1/QZN 2 with *p*-TsOH·NH_2_–Asp(OBzl)–OBzl/HCl·NH_2_–Glu(OCH_3_)–OCH_3_ was carried out using EDCI/HOBt as the coupling agent and NMM as the base to obtain 1, 2, 15 and 16. Next, these conjugates were refluxed with an excess of hydrazine hydrate to obtain the corresponding bis-hydrazides of the quinazolinones, 3, 4, 17 and 18. These bis-hydrazides were allowed to react with various substituted aldehydes to obtain the bis-hydrazones of the quinazolinone derivatives, 5–14 and 19–28. All the compounds were obtained in good yields. The structures of all the newly synthesized compounds were confirmed by IR, ^1^H-NMR, ^13^C-NMR and mass spectral analyses. The conjugation of the quinazolinones with the amino acids was confirmed by the appearance of an –NH peak at *δ* 8.54–8.15 and the absence of a COOH signal at *δ* 12.25 in the ^1^H NMR spectrum. In the IR spectra, bands appeared at 3310 cm^−1^ (–NH_2_) and 3217 cm^−1^ (–NH) and in the PMR spectra, peaks appeared at *δ* 4.12–3.26 (–NH_2_) and 9.11–8.88 (–NH) which indicated the conversion of an ester into a hydrazide. The formation of the hydrazones (–N=CH) was confirmed by the presence of an absorption band at 1612–1630 cm^−1^ and a peak at *δ* 8.62–8.30. The presence of all requisite peaks and absence of extraneous peaks in the PMR and CMR spectra confirm the structures. Furthermore, the mass values obtained were in good agreement with the structures assigned (spectral data are provided in the ESI†).

### 2.2. Biology

#### 2.2.1. Antioxidant activity

The *in vitro* antioxidant activity of the synthesized compounds was evaluated with DPPH,^29^ DMPD,^30,31^ and ABTS^32^ radical scavenging assays. The values were determined in terms of the IC_50_, the concentration at which 50% of the radicals were scavenged, and were calculated to evaluate the antioxidant activity. The IC_50_ values were compared with those of the standards: ascorbic acid (AA) and gallic acid (GA). The results are tabulated in Table 1.

**Table 1:** Antioxidant activity and docking studies of the synthesized quinazolinone derivatives^a^

Entry	Antioxidant activity			Molecular docking studies with 2HCK protein			
	DPPH, IC_50_ (μg mL^−1^)	DMPD, IC_50_ (μg mL^−1^)	ABTS, IC_50_ (μg mL^−1^)	Docking score	H-bond interactions with AA^b^ residues	π-cation interactions	π–π stacking interactions
1	>300	280 ± 5.23	290 ± 3.26	−3.865	Arg 136	Arg 76	NF
2	260 ± 3.56	>300	>300	−4.047	NF	NF	NF
3	250 ± 2.21	240 ± 1.35	180 ± 1.65	−4.545	Ala 96	NF	NF
4	190 ± 1.40	>300	240 ± 1.32	−4.606	Ala 96, Asn 46	NF	NF
5	85 ± 0.95	100 ± 1.56	70 ± 1.45	−6.250	Asn 46	NF	NF
6	50 ± 0.45	55 ± 0.56	45 ± 1.20	−7.770	Ala 96, Asn 46	NF	NF
7	35 ± 0.65	40 ± 0.72	25 ± 0.95	−9.375	Asp 73, Asp 73, Asn 46	Arg 76	NF
8	120 ± 1.26	140 ± 2.36	150 ± 0.68	−5.066	Arg 76, Arg 136	NF	NF
9	180 ± 0.65	160 ± 0.65	145 ± 1.98	−5.983	Arg 136	NF	NF
10	70 ± 0.65	95 ± 1.67	100 ± 1.85	−6.193	NF	NF	NF
11	50 ± 1.01	55 ± 0.98	45 ± 0.89	−7.779	Arg 136	Arg 76	NF
12	25 ± 0.26	30 ± 0.65	40 ± 0.65	−9.007	Asp 73, Asp 73, Asn 46	NF	Arg 136
13	150 ± 3.26	135 ± 1.69	160 ± 1.98	−5.390	Arg 136, Asn 46	NF	NF
14	180 ± 2.10	170 ± 0.98	155 ± 1.69	−5.797	Arg 136, Asn 46	NF	NF
15	260 ± 3.65	>300	285 ± 2.65	−3.865	Arg 136, Gly 77	NF	NF
16	>300	260 ± 3.45	>300	−4.188	NF	NF	NF
17	240 ± 2.98	225 ± 1.65	250 ± 3.56	−4.582	Ala 96, Asn 46, Gly 117	NF	NF
18	>300	245 ± 2.56	280 ± 2.45	−4.606	Ala 96, Gly 117	NF	NF
19	95 ± 0.95	80 ± 1.20	75 ± 0.56	−6.149	Arg 136	NF	NF
20	45 ± 0.88	55 ± 0.75	40 ± 0.45	−7.983	NF	NF	NF
21	30 ± 0.23	35 ± 0.98	35 ± 0.69	−9.256	Arg 136, Asp 49, Asp 49, Glu 42, Glu 42	Arg 76	Arg 76
22	150 ± 2.23	120 ± 2.45	135 ± 1.90	−5.416	Arg 136, Asn 46	NF	NF
23	200 ± 2.48	220 ± 1.65	205 ± 1.98	−5.129	NF	NF	NF
24	75 ± 1.45	80 ± 1.70	100 ± 1.65	−6.132	Arg 136	NF	NF
25	35 ± 0.68	45 ± 1.20	50 ± 1.95	−8.097	Glu 50	NF	NF
26	25 ± 0.24	30 ± 0.50	45 ± 0.64	−9.510	Arg 136, Asp 73, Asp73, Glu 42	Arg 76	NF
27	135 ± 1.98	150 ± 2.25	160 ± 1.40	−5.309	Asn 46, Glu 117	NF	NF
28	165 ± 0.65	140 ± 2.50	130 ± 2.60	−4.973	Arg 136	NF	NF
AA	50 ± 1.33	55 ± 2.26	45 ± 1.65	−6.499	Asp 73, Val 71	NF	NF
GA	60 ± 1.46	65 ± 1.26	50 ± 0.36	−6.397	Asp 73	NF	NF

a The values are the means of three determinations, the ranges of which are <5% of the mean in all cases.

b Amino acid; NF: not formed, AA = ascorbic acid, GA = gallic acid.

Most of the synthesized compounds showed antioxidant properties in all three radical scavenging assays. The conjugations of the QZN moiety with Asp (1 and 2) and Glu (15 and 16) showed antioxidant properties at higher concentrations which is in accordance with our earlier observations.^33^ Conversion of the esters into hydrazides (3, 4, 17 and 18) slightly improved the antioxidant potential, which may be due to the radical scavenging ability of NH–NH_2_ groups.^34^ The enhanced activity of the resultant hydrazones could be due to the presence of groups on the phenyl ring. Those compounds that lack substituents on the phenyl ring (5, 10, 19 and 24) showed moderate antioxidant activity with IC_50_ values ranging from 70–100 μg mL^−1^. Compounds with electron donating substituents like –OH and –OCH_3_ on the phenyl ring (6, 7, 11, 12, 20, 21, 25 and 26) exhibited excellent radical scavenging activity with IC_50_ values ranging from 25–55 μg mL^−1^ in all three antioxidant assays and even better results compared to the standards AA (45–55 μg mL^−1^) and GA (50–65 μg mL^−1^). In addition, the presence of OH slightly improved the antioxidant activity compared to that with OCH_3_. In contrast, compounds with electron withdrawing groups like –Cl and –NO_2_ on the phenyl ring (8, 9, 13, 14, 22, 23, 27 and 28) showed the lowest antioxidant activity with IC_50_ values ranging from 120–220 μg mL^−1^. On the basis of the above observation, it may be stated that compounds containing EDGs are excellent antioxidants,^35–37^ whereas the presence of EWGs decreases the antioxidant potential of the synthesized analogues.^22^ With respect to the length of the alkyl chain in the QZNs, there was no significance for the antioxidant potential. Replacement of Asp with Glu makes the antioxidant activity even better.

#### 2.2.2. Anti-inflammatory activity

The human erythrocyte suspension was used to evaluate the *in vitro* anti-inflammatory activity of the synthesized molecules by employing a literature known method.^38^ A substantial number of compounds exhibited excellent to moderate activity compared to the standards, indomethacin (IM) and ibuprofen (IP). The results of the IC_50_ measurements were determined and are tabulated in Table 2. The compounds with electron withdrawing groups (8, 9, 13, 14, 22, 23, 27 and 28) showed excellent activity with IC_50_ values of 45, 25, 50, 45, 50, 40, 45 and 35 μg mL^−1^, respectively, which are more potent than those of indomethacin (55 μg mL^−1^) and ibuprofen (50 μg mL^−1^). Besides this, compounds with electron donating substituents like OH and OCH_3_ (6, 7, 11, 12, 20, 21, 25 and 26) or without any substituents (5, 10, 19 and 24) showed moderate activity. This clearly indicates that the compounds with electron withdrawing groups (Cl, NO_2_) on the phenyl ring are better anti-inflammatory agents.^39^ Furthermore, NO_2_ which is a powerful EWG is better at enhancing the anti-inflammatory activity than Cl.

**Table 2:** Anti-inflammatory activity and docking studies of the synthesized quinazolinone derivatives^a^

Entry	Anti-inflammatory activity, IC_50_ (μg mL^−1^)	Molecular docking studies with 1CX2 protein				
		Docking score	H-bond interactions with AA^b^ residues	Electrostatic forces of attraction	π-cation interactions	π–π stacking interactions
1	270 ± 4.25	−4.164	Lys 118	NF	NF	NF
2	>300	−4.199	Lys 118	NF	NF	Phe 30
3	225 ± 2.69	−3.905	Lys 118	NF	NF	NF
4	>300	−4.068	Lys 118	NF	NF	Phe 30
5	100 ± 2.60	−4.678	NF	NF	NF	Phe 30, Phe 30
6	75 ± 1.50	−4.879	Asp, 87, Lys 118	NF	NF	NF
7	80 ± 2.06	−4.976	Ala 161, Asp 90, Asp 90, Asp 120, Asp 120, Lys 118	NF	NF	Phe 30
8	45 ± 0.25	−8.576	NF	NF	NF	Phe 30
9	25 ± 0.56	−8.908	Cys 20, Lys 118, Lys 118	Asp 13, Asp 87, Lys 118, Lys 118	NF	Tyr 34
10	120 ± 1.40	−4.810	Lys 118, Asp 87	NF	NF	Phe 30
11	80 ± 1.50	−4.759	NF	NF	NF	NF
12	65 ± 1.60	−4.719	Ala 161, Asp 120, Asp 120, Glu 32, Glu 32,	NF	Lys 118	Phe 30
13	50 ± 2.95	−7.810	Asp 87	NF	NF	His 126, Phe 30
14	45 ± 1.28	−8.027	Asp 87, Cys 20, Lys 118, Ser 88	Asp 13, Asp 87, Asp 90, Lys 118	NF	NF
15	270 ± 2.90	−3.799	Lys 118, Gly 17	NF	NF	NF
16	>300	−3.850	Lys 118	NF	NF	NF
17	240 ± 3.40	−4.116	Asp 120, Lys 118, Lys 162	NF	Lys 118	NF
18	260 ± 2.75	−3.630	Asp 87, Lys 118, Thr 127	NF	NF	Phe 30
19	150 ± 2.35	−4.378	NF	NF	NF	NF
20	80 ± 0.54	−4.646	NF	NF	Lys 118	Phe 30
21	95 ± 0.45	−4.506	Asp 87, Asp 87, Asp 124, Lys 118, Lys 162, Lys 162	NF	NF	NF
22	50 ± 1.20	−7.843	Asp 87	NF	NF	Phe 30
23	40 ± 0.64	−8.023	Asp 87, Asp 87, Lys 118	Asp 13, Lys 118, Lys 162	NF	Phe 30
24	115 ± 0.75	−4.378	Asp 87	NF	NF	His 126
25	75 ± 0.80	−4.482	Asp 87	NF	Lys 118	Phe 30
26	90 ± 2.20	−4.472	Asp 87, Asp 87, Asp 90, Ser 88	NF	NF	Phe 30
27	45 ± 0.90	−8.143	Asp 87, Lys 118	NF	NF	Phe 30
28	35 ± 0.64	−7.928	Asp 87	Tyr 34	Lys 118	Tyr 34
IM	55 ± 2.01	−5.074	NF	NF	Lys 118	Phe 30
IP	50 ± 1.26	-	-	-	-	-

a The values are the means of three determinations, the ranges of which are <5% of the mean in all cases.

b Amino acid; NF: not formed; ‘-’: no activity/not analyzed, IM = indomethacin, IP = ibuprofen.

#### 2.2.3. Antimicrobial activity

The efficacy of the synthesized compounds as antimicrobials was tested in antibacterial^40^ studies against different strains of pathogen using both Gram negative bacteria, namely *Escherichia coli* (*E. coli*), and Gram positive bacteria, *Staphylococcus aureus* (*S. aureus*), and in antifungal^41^ studies against *Fusarium moniliforme* (*F. moniliforme*) and *Aspergillus niger* (*A. niger*). The results obtained as the zone of inhibition (mm) are presented in Table 3. Streptomycin (SM) and bavistin (BS) served as the standard drugs for the antibacterial and antifungal studies, respectively.

**Table 3:** Antimicrobial activity and molecular docking studies of the synthesized analogues with 2VF5

Entry	Antibacterial activity^a^ (mm)		Antifungal activity^a^ (mm)		Molecular docking studies with 2VF5		
					Docking score	Hydrogen bond interactions with amino acid residues	*π-cation interactions/^#^electrostatic forces of attraction
	*E. coli*	*S. aureus*	*F. moniliforme*	*A. niger*			
1	04 ± 0.21	02 ± 0.32	05 ± 0.12	02 ± 0.41	−6.150	Ala 602, Glu 488, Ser 401	*Lys 603
2	03 ± 0.38	NA	02 ± 0.35	NA	−6.196	Glu 488, Gly 301, Ser 401	NF
3	09 ± 0.65	07 ± 0.65	06 ± 0.65	04 ± 0.68	−7.990	Ala 602, Lys 603, Ser 347, Gln 348, Gln 348, Ser 303, Leu 346, Cys 300	NF
4	07 ± 0.45	NA	05 ± 0.38	03 ± 0.12	−7.804	Thr 302, Ser 401, Leu 346, Cys 300, Gln 348, Thr 352, Ser 303, Ser 347	NF
5	14 ± 0.32	10 ± 0.52	11 ± 0.42	09 ± 0.25	−8.203	Ala 602, Val 399, Lys 603, Gln 348, Ser 401, Ser 347	NF
6	16 ± 0.54	14 ± 0.16	15 ± 0.14	18 ± 0.54	NF	NF	NF
7	28 ± 0.32	25 ± 0.25	26 ± 0.28	24 ± 0.32	−12.238	Asp 354, Asp 354, Glu 488, Ala 602, Ser 401, Gln 348, Ser 303, Gly 301, Thr 352	NF
8	18 ± 0.18	16 ± 0.45	17 ± 0.36	15 ± 0.65	−8.597	Glu 488	NF
9	21 ± 0.23	19 ± 0.32	23 ± 0.23	24 ± 0.23	−10.017	Asp 354, Ala 602, Ala 602	^#^Asp 354, ^#^Asp 354
10	10 ± 0.56	09 ± 0.23	11 ± 0.65	12 ± 0.45	NF	NF	NF
11	16 ± 0.36	15 ± 0.55	17 ± 0.11	18 ± 0.78	NF	NF	NF
12	26 ± 0.65	24 ± 0.85	25 ± 0.45	27 ± 0.28	−10.553	Asp 354, Asp 354, Val 399, Cys 300, Ser 347, Ser 349, Ala 602, Asn 600	NF
13	18 ± 0.12	17 ± 0.35	19 ± 0.26	20 ± 0.45	NF	NF	NF
14	22 ± 0.26	20 ± 0.38	21 ± 0.65	22 ± 0.22	−9.835	Lys 603, Ser 303, Asp 354	^#^Asp 354, ^#^Asp 354
15	02 ± 0.35	NA	05 ± 0.32	01 ± 0.18	−5.966	Ala 602, Ala 602, Val 605, Thr 352, Ser 347, Ser 349, Gln 348, Ser 303	NF
16	NA	01 ± 0.08	NA	04 ± 0.26	−5.816	Ala 602, Thr 302, Ser 349, Ser 347	NF
17	04 ± 0.21	03 ± 0.21	05 ± 0.33	08 ± 0.21	−7.199	Ala 602, Ala 602, Leu 346, Cys 300, Gln 348, Ser 347, Lys 603, Lys 603	NF
18	05 ± 0.65	NA	07 ± 0.29	NA	−7.170	Ala 602, Ala 602, Cys 300, Ser 347, Ser 349, Gln 348, Ser 401, Ser 401, Ser 303	NF
19	14 ± 0.35	11 ± 0.65	13 ± 0.26	14 ± 0.29	−8.083	Gln 348, Val 399, Thr 302, Ala 602, Ser 401	NF
20	18 ± 0.16	16 ± 0.19	15 ± 0.32	16 ± 0.11	−8.317	Ala 602, Lys 603, Ser 303	NF
21	28 ± 0.22	26 ± 0.35	25 ± 0.24	24 ± 0.26	−9.339	Thr 352, Ser 303, Asn 600, Ala 602, Asn 305, Gly 301	NF
22	19 ± 0.32	20 ± 0.17	18 ± 0.35	17 ± 0.56	−8.432	Thr 352, Lys 603, Thr 302, Val 399, Ala 602	NF
23	22 ± 0.56	24 ± 0.29	21 ± 0.65	23 ± 0.36	−8.969	Thr 302, Gln 348, Ser 303, Ser 347, Thr 352	^#^Glu 488
24	13 ± 0.25	10 ± 0.56	15 ± 0.36	14 ± 0.33	NF	NF	NF
25	16 ± 0.32	17 ± 0.44	18 ± 0.14	15 ± 0.38	NF	NF	NF
26	26 ± 0.35	22 ± 0.23	24 ± 0.36	25 ± 0.36	−9.282	Asn 305, Thr 352, Gln 348	NF
27	19 ± 0.26	16 ± 0.66	19 ± 0.56	20 ± 0.11	NF	NF	NF
28	24 ± 0.12	23 ± 0.33	24 ± 0.45	22 ± 0.65	NF	NF	NF
SM	15 ± 0.36	13 ± 0.69	—	—	−9.517	Gly 301, Asp 354, Asp 354, Cys 300, Thr 302, Ala 602, Ala 602, Glu 488	^#^Glu 488
BS	—	—	14 ± 0.65	13 ± 0.36	NF	NF	NF

a The values are the means of three determinations, the ranges of which are <5% of the mean in all cases, NF = not formed, SM = streptomycin, BS = bavistin.

Most of the synthesized compounds showed promising antimicrobial activity with few exceptions. The results of the antimicrobial studies showed that all the analogues exhibited the same trend for both antibacterial and antifungal activity. The quinazolinone–amino acid conjugates (1, 2, 15 and 16) exhibited a much smaller zone of inhibition. When C-terminal benzyl ester/methyl ester groups were converted into hydrazides (4, 5, 17 and 18), there was a slight enhancement in the activity. This may be due to the increase in the polarity of the compounds, which would help the molecule to penetrate more through the cell membrane of microbes and thereby inactivate them.^20^ There was a drastic enhancement in the activity when the hydrazides were converted into hydrazones and we observed that the nature of the substituents present on the phenyl ring affected the biological activity of the compounds to a greater extent.

Among the bis-hydrazones, the compounds with hydroxyl groups (7, 12, 21 and 26) and nitro groups (9, 14, 23 and 28) on the phenyl ring showed potent antimicrobial activity compared to the standard drugs. Compounds bearing methoxy (6, 11, 20 and 25) and chloro (8, 13, 22 and 27) groups showed moderate antibacterial properties. Those compounds (5, 10, 19 and 24) which did not have any substituents on the phenyl ring showed the lowest activity. Thus the order of activity based on the groups attached to the phenyl ring of hydrazone derivatives was found to be OH > NO_2_ > Cl > OCH_3_ > H. An increase in the alkyl chain length of quinazolinone caused a slight decrease in the antimicrobial activity which is in good agreement with our earlier report.^20^ Replacement of Asp with Glu resulted in a slight decrease in the antimicrobial properties which may be attributed to the increase in hydrophobicity caused by the longer carbon chain in Glu.

### 2.3. Molecular docking studies

The results of the molecular docking studies of the title compounds with respect to the antioxidant, anti-inflammatory and antimicrobial properties are presented below.

**Fig. 2:**
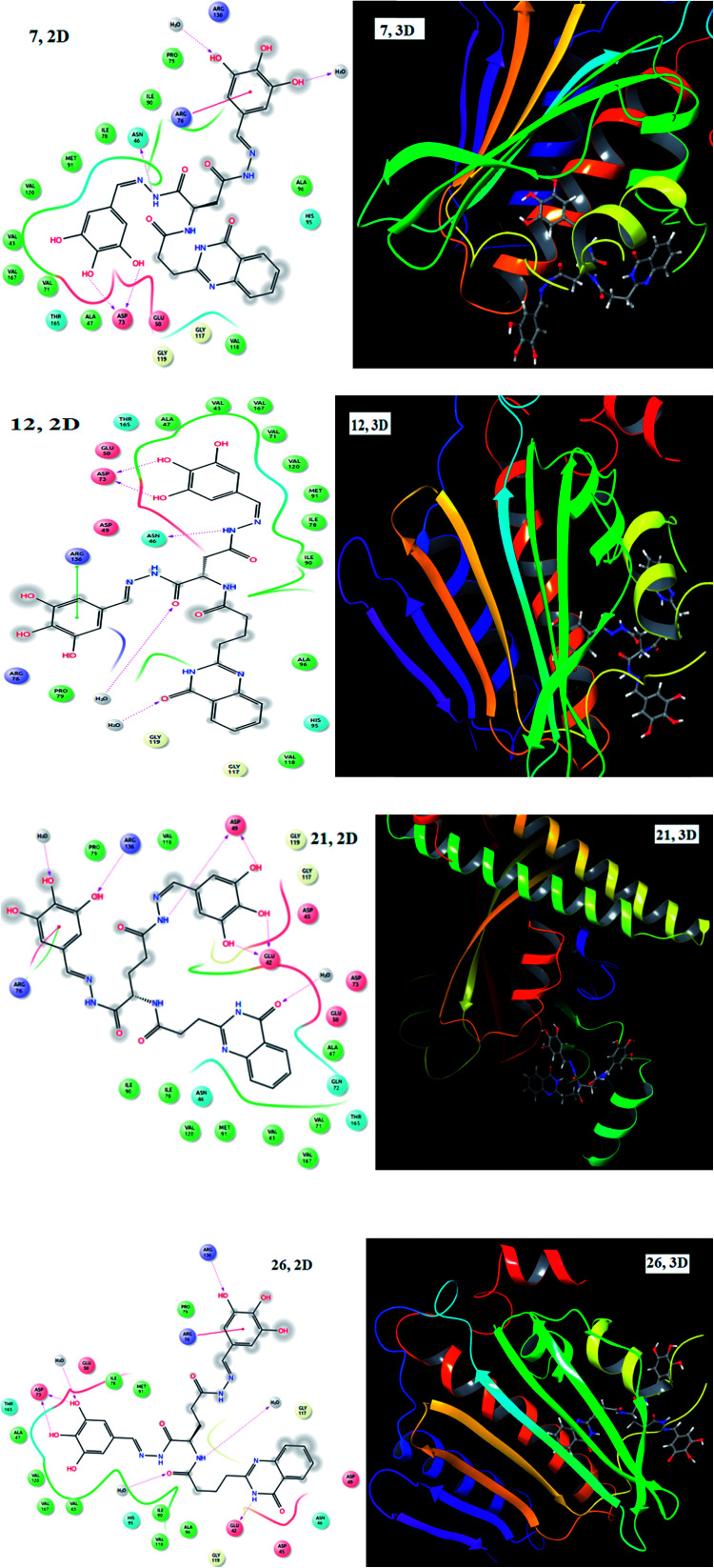
2D and 3D images of compounds 7, 12, 21 and 26 with 2HCK.

Molecular docking was performed on the active site of tyrosine kinase (PDB ID: 2HCK) with the synthesized ligands (1–28) in order to determine the possible binding interactions of highly potent molecules. Most of the compounds showed good docking scores and potent interactions with different amino acid residues and the results are tabulated in Table 1. Among the series of compounds, those possessing EDGs, especially hydroxyl groups (7, 12, 21 and 26), gave the highest docking scores. The binding interactions (2D and 3D) of 7, 12, 21 and 26 are displayed in Fig. 2. Compound 7 showed hydrogen bond interactions with Asp 73, Asp 73 and Asn 46 and π-cation interactions with Arg 76 and compound 12 showed hydrogen bond interactions with Asp 73, Asp 73, and Asn 46 and π–π stacking interactions with Arg 136. Compound 21 showed hydrogen bond interactions with Arg 136, Asp 49, Asp 49, Glu 42 and Glu 42 and π-cation and π–π stacking interactions with Arg 76 and compound 26 exhibited hydrogen bond interactions with Arg 136, Asp 73, Asp 73 and Glu 42 and π-cation interactions with Arg 76. These molecules showed the highest docking scores because of the involvement of hydroxyl groups on the phenyl ring in hydrogen bond interactions.

**Fig. 3:**
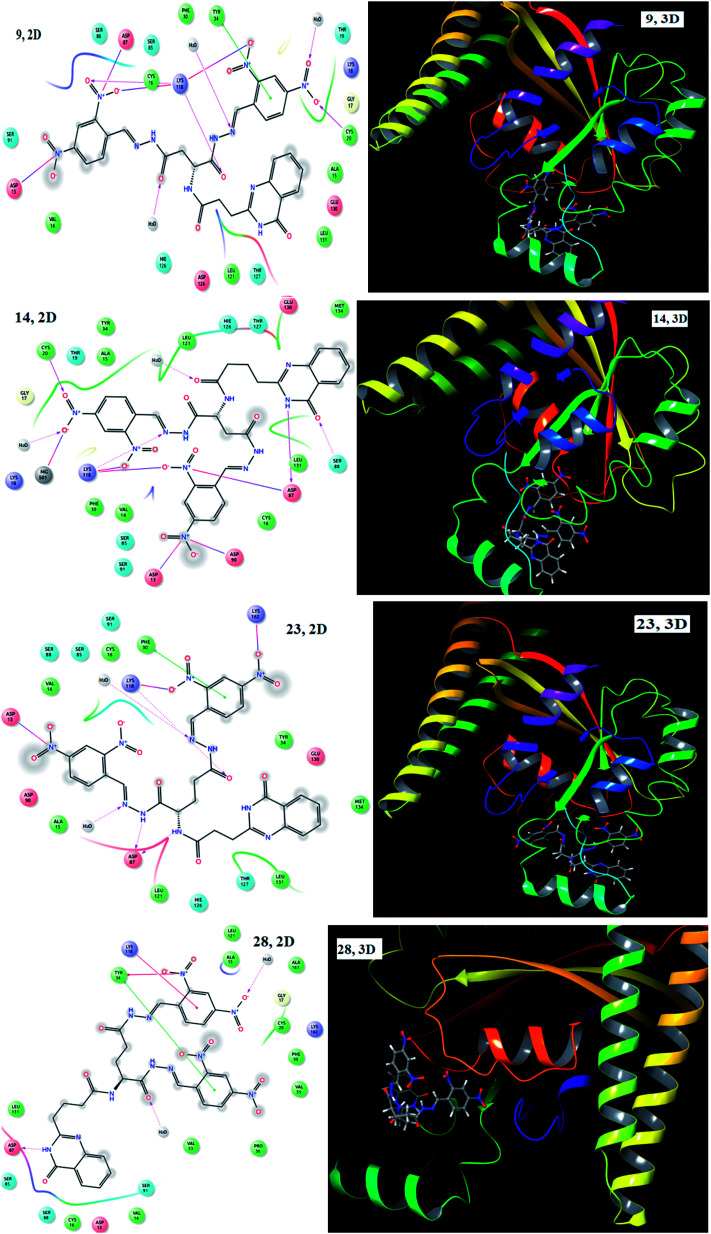
2D and 3D images of compounds 9, 14, 23 and 28 with 1CX2.

To rationalize the anti-inflammatory potential of the synthesized compounds (1–28), we conducted docking studies on the crystal structure of cyclooxygenase-2 (PDB ID: 1CX2). Most of the compounds exhibited a good docking score and different kinds of interaction with amino acid residues and these are tabulated in Table 2. Analogues with EWGs, particularly with NO_2_ (9, 14, 23 and 28) on the phenyl ring, displayed good interactions with amino acid residues and had the highest docking scores. Molecular docking studies provided an interaction map of cyclooxygenase-2 with 9, 14, 23 and 28 and the results are presented in Fig. 3. Compound 9 showed hydrogen bond interactions with Cys 20, Lys 118 and Lys 118, electrostatic forces of attraction with Asp 13, Asp 87, Lys 118 and Lys 188 and π–π stacking interactions with Tyr 34; and compound 14 showed hydrogen bond interactions with Asp 87, Cys 20, Lys 118 and Ser 88 and electrostatic interactions with Asp 13, Asp 87, Asp 90, Lys 118 and Lys 118. Meanwhile, compound 23 displayed hydrogen bond interactions with Asp 87, Asp 87 and Lys 118, electrostatic interactions with Asp 13, Lys 118 and Lys 162 and π–π stacking interactions with Phe 30, whereas compound 28 showed hydrogen bond interactions with Asp 87, π-cation interactions with Lys 118 and Tyr 34 and π–π stacking interactions with Tyr 34. The NO_2_ groups present on the phenyl ring involved in electrostatic forces of attraction and π-cation interactions led to an enhanced docking score.

**Fig. 4:**
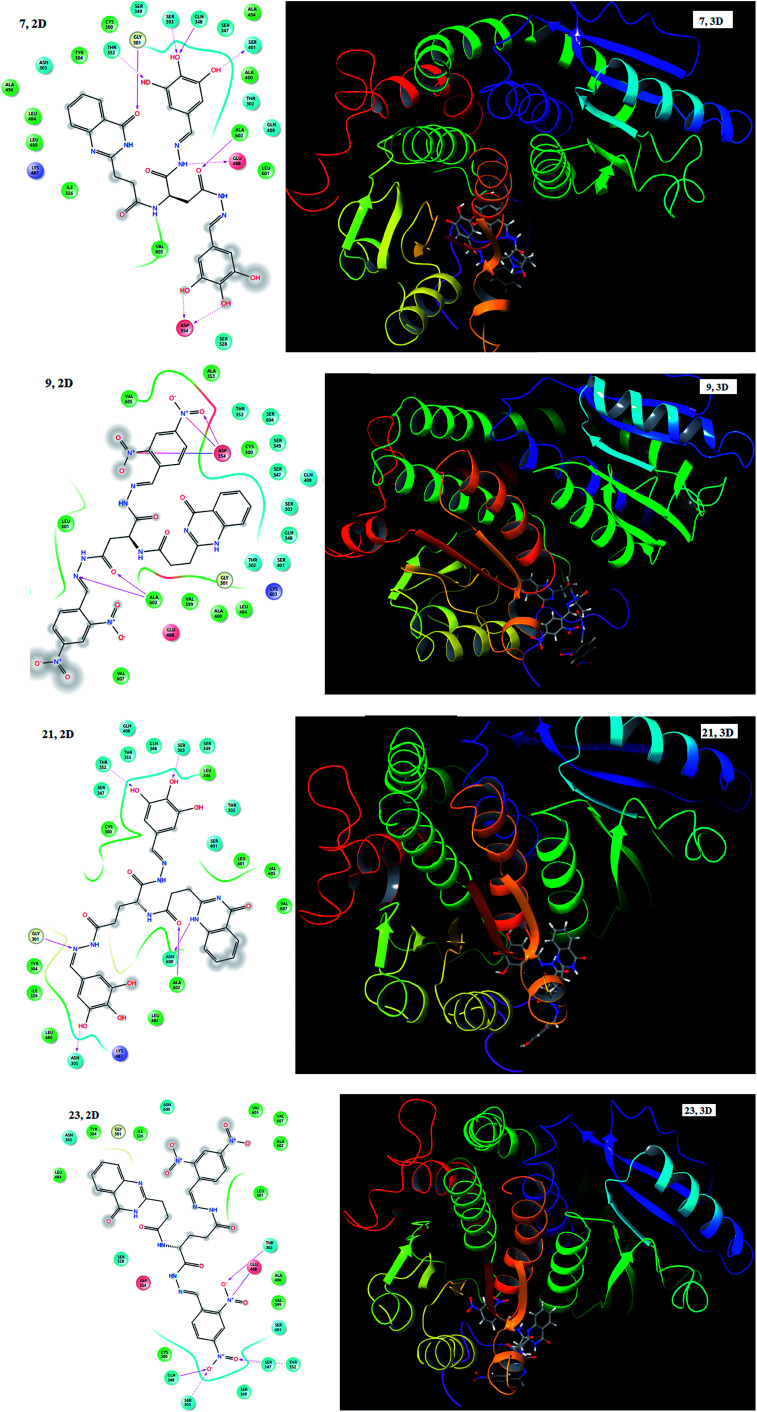
2D and 3D images of compounds 7, 9, 21 and 23 with 2VF5 protein.

In order to gain insight into the exact binding location of the ligands with the protein, all the synthesized molecules (1–28) including the standards were subjected to molecular docking with the active site of GlcN-6-P synthase (PDB ID: 2VF5). They showed good binding interactions with the surrounding amino acid residues. The docking score and the interacting amino acids are tabulated in Table 3. Most of the synthesized compounds exhibited well established bonds with one or more amino acids in the receptor active pocket of 2VF5 protein. The potential of the compounds as antimicrobial agents was determined based on the docking scores. The docking scores with 2VF5 protein ranged from −12.238 to −5.816 and the highest negative value indicates the best docked ligand to the targeted site. The 2D and 3D images of compounds 7, 9, 21 and 23 are shown in Fig. 4. Aspartic acid linked hydrazones (7) showed hydrogen bond interactions with Asp 354, Asp 354, Glu 488, Ala 602, Ser 401, Gln 348, Ser 303, Gly 301 and Thr 352 and compound 9 showed hydrogen bond interactions with Asp 354, Ala 602 and Ala 602 and electrostatic forces of interaction with Asp 354 and Asp 354. On the other hand, glutamic acid linked hydrazones (21) displayed hydrogen bond interactions with Thr 352, Ser 303, Asn 600, Ala 602, Asn 305 and Gly 301 and compound 23 showed hydrogen bond interactions with Thr 302, Gln 348, Ser 303, Ser 347 and Thr 352 and electrostatic forces of interaction with Glu 488. The involvement of the hydroxyl groups in hydrogen bond interactions and the nitro groups in electrostatic forces of attraction enhanced the docking scores of these analogues.

## 3. Conclusions

In summary, we have developed a novel class of antioxidant, anti-inflammatory and antimicrobial agent with quinazolinone-hydrazones linked *via* Asp and Glu as connectors. In addition, molecular docking studies were performed on all the synthesized compounds. The correlation between molecular docking studies and biological assays suggested that compounds 6, 7, 11, 12, 20, 21, 25 and 26 with electron donating groups (OH, OCH_3_) exhibited stronger radical scavenging activity than ascorbic acid and gallic acid. Compounds 8, 9, 13, 14, 22, 23, 27 and 28 with electron withdrawing groups (Cl, NO_2_) demonstrated better *in vitro* anti-inflammatory activity than indomethacin and ibuprofen. The compounds with electron donating hydroxyl groups (7, 12, 21 and 26) and electron withdrawing nitro groups (9, 14, 23 and 28) exhibited potent antimicrobial properties among this series of compounds.

## Conflicts of interest

There are no conflicts to declare.

## Supplementary Material

RA-008-C8RA00531A-s001
