# Supplementary material for: A correlation study of biological activity and molecular docking of Asp and Glu linked bis-hydrazones of quinazolinones
Source: RSC Adv. 2018 Mar 16;8(19):10644–53. doi: 10.1039/c8ra00531a (PMC9078910; doi:10.1039/c8ra00531a)
Supplement: RA-008-C8RA00531A-s001 [file RA-008-C8RA00531A-s001.pdf]

Electronic Supplementary Material (ESI) for RSC Advances.
This journal is © The Royal Society of Chemistry 2018

Supplementary data

A correlation study of biological activity and molecular docking of Asp and Glu linked *bis*-hydrazones of quinazolinones

H. K. Kumara, R. Suhas, D. M. Suyoga Vardhan, M. Shobha and D. Channe Gowda*

Department of Studies in Chemistry, University of Mysore, Manasagangotri, Mysuru - 570 006, Karnataka, India

*Correspondence: D. Channe Gowda
E-mail address: dchannegowda@yahoo.co.in
Tel.: +91 821 2419664

Experimental

General

The amino acids used were of *L*-configuration unless otherwise mentioned. Different substituted aldehydes and protected amino acids were purchased from Sigma Aldrich (Bengaluru, India). Hydrazine hydrate, EDCI, HOBt and NMM were purchased from Merck Pvt. Ltd., (Mumbai, India). All solvents and reagents used for the synthesis were of analytical grade. Progress of the reaction was monitored by TLC using silica gel 60 F_{254} aluminium plates with the solvent system comprising chloroform/methanol/acetic acid in the ratio 98:02:03 (R_f^a) and 95:05:03 (R_f^b). The compounds on the TLC plates were identified by using UV lights. Melting points were determined on a Superfit melting point apparatus (India) and are uncorrected. FT-IR was performed using a Jasco spectrometer (Japan) using nujol media. ^{1}H NMR (400 MHz) and ^{13}C NMR (100 MHz) spectra were recorded on an Agilent NMR instrument (USA) using DMSO-d_6 as a solvent. High resolution mass spectroscopic analysis was performed on a Bruker MicroTOF QII mass spectrometer in positive mode.

Synthesis

Synthesis of QZN1-Asp(OBzl)-OBzl (**1**), QZN2-Asp(OBzl)-OBzl (**2**), QZN1-Glu(OMe)-OMe (**15**), QZN2-Glu(OMe)-OMe (**16**)

To a stirred solution of QZN 1 (2.18 g, 10 mmol) and QZN 2 (2.32 g, 10 mmol) separately in DMF (10 mL/g of compound) and cooled to 0 °C was added NMM (1.09 mL, 10 mmol). EDCI (2.30 g, 10 mmol) was added under stirring while maintaining the temperature at 0 °C. After stirring the reaction mixture for 10 minutes added HOBt (1.53 g, 10 mmol) and allowed to stir for an additional 10 minutes. Then a pre-cooled solution of aspartic acid dibenzyl ester *p*-toluene sulfonate salt (4.85 g, 10 mmol)/glutamic acid dimethyl ester hydrochloride salt (2.11 g, 10 mmol) and NMM (1.09 mL, 10 mmol) in DMF (10 mL/g of compound) was added slowly. After 20 minutes, pH of the solution was adjusted to 8 by the addition of NMM and the reaction mixture was stirred overnight at room temperature. DMF was removed under reduced pressure and the residual DMF solution was poured into about 500 mL ice-cold water and the resulted precipitate was filtered and washed with diethyl ether and dried to get desired compounds **1, 2, 15** and **16**.

Dibenzyl 2-(3-(4-oxo-3,4-dihydroquinazolin-2-yl)propanamido)succinate (1):

Yield 94.58%, white solid, R_f^a = 0.66, R_f^b = 0.76, m.p. 115-117 °C, IR KBr (cm^{-1}): 3235, 1760, 1655; ^{1}H NMR (DMSO-d_6, 400 MHz) δ ppm: 12.15 (s, 1H, Het-NH), 8.56-8.54 (d, 1H, $^\alpha$NH-Asp), 8.07-7.29 (m, 14H, Ar-H), 5.06 (s, 2H, Benzyl-CH_2), 5.04 (s, 2H, Benzyl-CH_2), 4.77-4.72 (q, 1H, $^\alpha$CH), 2.92-2.84 (t, 2H, CH_2), 2.82-2.75 (d, 2H, CH_2-Asp), 2.64-2.68 (t, 2H, CH_2); ^{13}C NMR (DMSO-d_6, 100 MHz) δ ppm: 170.97, 170.66, 170.23, 162.08, 156.90, 149.23, 136.30, 136.19, 134.61, 218.83, 128.82, 128.46, 128.44, 128.38, 128.32, 128.11, 127.20, 126.35, 126.14, 121.39, 66.69, 66.31, 49.17, 36.36, 31.64, 30.00; HRMS m/z: 514.2690 (M+1)

Dibenzyl 2-(4-(4-oxo-3,4-dihydroquinazolin-2-yl)butanamido)succinate (2):

Yield 95.96%, white solid, R_f^a = 0.54, R_f^b = 0.64, m.p. 109-111 °C, IR KBr (cm^{-1}): 3230, 1745, 1645; ^{1}H NMR (DMSO-d_6, 400 MHz) δ ppm: 12.14 (s, 1H, Het-NH), 8.55-8.53 (d, 1H, $^\alpha$NH-Asp), 8.08-7.30 (m, 14H, Ar-H), 5.05 (s, 2H, Benzyl-CH_2), 5.03 (s, 2H, Benzyl-CH_2), 4.76-4.70 (q, 1H, $^\alpha$CH), 2.94-2.85 (t, 2H, CH_2), 2.80-2.74 (d, 2H, CH_2-Asp), 2.66-2.69 (t, 2H, CH_2), 1.98-1.94 (m, 2H, CH_2); ^{13}C NMR (DMSO-d_6, 100 MHz) δ ppm: 170.94, 170.43, 170.12, 162.32, 156.85, 149.20, 136.56, 136.18, 134.85, 218.95, 128.65, 128.53, 128.40, 128.35, 128.30, 128.09, 127.28, 126.85, 126.28, 121.40, 66.80, 66.26, 49.25, 36.28, 30.65, 29.85, 23.05; HRMS m/z: 528.2316 (M+1)

Dimethyl 2-(3-(4-oxo-3,4-dihydroquinazolin-2-yl)propanamido)pentanedioate (15):

Yield 95.65%, white solid, R_f^a = 0.65, R_f^b = 0.76, m.p. 165-167 °C, IR KBr (cm^{-1}): 3252, 1752, 1648; ^{1}H NMR (DMSO-d_6, 400 MHz) δ ppm: 12.05 (s, 1H, Het-NH), 8.15 (d, 1H, $^\alpha$NH-Glu),

8.05-7.42 (m, 4H, Ar-H), 4.28-4.22 (q, 1H, $^{\alpha}$CH), 3.58 (s, 3H, OCH_3), 3.55 (s, 3H, OCH_3), 2.60-2.58 (t, 2H, CH_2), 2.37-2.30 (t, 2H, CH_2-Glu), 2.20-2.17 (t, 2H, CH_2), 1.91-1.78 (m, 2H, CH_2-Glu); ^{13}C NMR (DMSO-d_6, 100 MHz) δ ppm: 172.86, 172.72, 172.56, 162.28, 157.40, 149.45, 134.98, 127.20, 126.65, 126.45, 121.32, 52.65, 51.95, 51.45, 34.56, 34.20, 30.75, 26.50; HRMS m/z: 375.2065 (M+1)

Dimethyl 2-(4-(4-oxo-3,4-dihydroquinazolin-2-yl)butanamido)pentanedioate (16):

Yield 94.20%, white solid, R_f^a = 0.69, R_f^b = 0.77, m.p. 160-162 °C, IR KBr (cm^{-1}): 3245, 1768, 1655; ^{1}H NMR (DMSO-d_6, 400 MHz) δ ppm: 12.07 (s, 1H, Het-NH), 8.16 (d, 1H, $^{\alpha}$NH-Glu), 8.06-7.40 (m, 4H, Ar-H), 4.27-4.21(q, 1H, $^{\alpha}$CH), 3.59 (s, 3H, OCH_3), 3.55 (s, 3H, OCH_3), 2.61-2.57 (t, 2H, CH_2), 2.36-2.32 (t, 2H, CH_2-Glu), 2.21-2.18 (t, 2H, CH_2), 1.98-1.94 (m, 2H, CH_2), 1.92-1.77 (m, 2H, CH_2-Glu); ^{13}C NMR (DMSO-d_6, 100 MHz) δ ppm: 172.97, 172.66, 172.43, 162.20, 157.37, 149.34, 134.62, 127.26, 126.35, 126.10, 121.33, 52.28, 51.78, 51.54, 34.53, 34.18, 30.12, 26.52, 23.04; HRMS m/z: 390.1309 (M+1)

Synthesis of QZN1-Asp($NHNH_2$)-$NHNH_2$ (**3**), QZN2-Asp($NHNH_2$)- $NHNH_2$ (**4**), QZN1-Glu($NHNH_2$)- $NHNH_2$ (**17**), QZN2-Glu($NHNH_2$)- $NHNH_2$ (**18**)

To a separate solution of **1** (4.50 g, 8.76 mmol), **2** (4.62 g, 8.76 mmol), **15** (3.28 g, 8.76 mmol), and **16** (3.41 g, 8.76 mmol) in ethanol (40 mL), hydrazine hydrate (1.27 mL, 26.28 mmol) was added slowly using a dropping funnel. The reaction mixture was refluxed for 16 h on an oil bath and the completion of the reaction was monitored by TLC. The solvent was removed under reduced pressure and cooled by adding ice cold water. The resulting precipitate was filtered, washed with cold water and recrystallized from ethanol to get the desired compounds **3, 4, 17** and **18**.

N-(1,4-Dihydrazinyl-1,4-dioxobutan-2-yl)-3-(4-oxo-3,4-dihydroquinazolin-2-yl) propanamide (3):

Yield 71.09%, white solid, R_f^a = 0.12, R_f^b = 0.23, m.p. 201-203 °C, IR KBr (cm^{-1}): 3320, 3250, 1765, 1634; ^{1}H NMR (DMSO-d_6, 400 MHz) δ ppm: 11.97 (s, 1H, Het-NH), 8.97 (s, 1H, NH-hydrazide), 8.88 (s, 1H, NH-hydrazide), 8.04-8.02 (d, 1H, $^{\alpha}$NH-Asp), 8.04-7.39 (m, 4H, Ar-H), 4.56-4.50 (q, 1H, $^{\alpha}$CH), 3.28 (s, 4H, NH_2), 2.82-2.65 (t, 2H, CH_2), 2.63-2.56 (t, 2H, CH_2), 2.46-2.27 (d, 2H, CH_2-Asp); ^{13}C NMR (DMSO-d_6, 100 MHz) δ ppm: 171.38, 170.44, 169.12, 162.11, 157.20, 149.13, 134.64, 127.17, 126.35, 126.10, 121.30, 49.13, 36.32, 32.03, 30.20; HRMS m/z: 362.2128 (M+1)

N-(1,4-Dihydrazinyl-1,4-dioxobutan-2-yl)-4-(4-oxo-3,4-dihydroquinazolin-2-yl)butanamide (4):

Yield 72.21%, white solid, R_f^a = 0.13, R_f^b = 0.24, m.p. 224-226 °C, IR KBr (cm^{-1}): 3310, 3225, 1745, 1655; ^1H NMR (DMSO-d_6, 400 MHz) δ ppm: 11.94 (s, 1H, Het-NH), 8.95 (s, 1H, NH-hydrazide), 8.80 (s, 1H, NH-hydrazide), 8.03-8.01 (d, 1H, $^{\alpha}$NH-Asp), 8.03-7.29 (m, 4H, Ar-H), 4.60-4.55 (q, 1H, $^{\alpha}$CH), 3.48 (s, 4H, NH_2), 2.90-2.68 (t, 2H, CH_2), 2.62-2.55 (t, 2H, CH_2), 2.44-2.25 (d, 2H, CH_2-Asp), 2.08-1.96 (m, 2H, CH_2); ^{13}C NMR (DMSO-d_6, 100 MHz) δ ppm: 171.38, 170.44, 169.12, 162.11, 157.20, 149.13, 134.64, 127.17, 126.35, 126.10, 121.30, 49.13, 36.32, 32.03, 30.20, 24.05; HRMS m/z: 376.1834 (M+1)

N-(1,5-Dihydrazinyl-1,5-dioxopentan-2-yl)-3-(4-oxo-3,4-dihydroquinazolin-2-yl) propanamide (17):

Yield 72.85%, white solid, R_f^a = 0.13, R_f^b = 0.22, m.p. 210-212 °C, IR KBr (cm^{-1}): 3352, 3295, 1758, 1634; ^1H NMR (DMSO-d_6, 400 MHz) δ ppm: 12.12 (s, 1H, Het-NH), 9.08 (s, 1H, NH-hydrazide), 8.95 (s, 1H, NH-hydrazide), 8.06-8.04 (d, 1H, $^{\alpha}$NH-Glu), 7.95-7.39 (m, 4H, Ar-H), 4.20-4.15 (q, 1H, $^{\alpha}$CH), 4.15 (s, 4H, NH_2), 2.62-2.54 (t, 2H, CH_2), 2.22-2.16 (t, 2H, CH_2-Glu), 2.05-1.90 (t, 2H, CH_2), 1.85-1.62 (m, 2H, CH_2-Glu); ^{13}C NMR (DMSO-d_6, 100 MHz) δ ppm: 172.25, 171.38, 171.24, 162.45, 157.50, 149.42, 134.78, 127.30, 126.25, 126.25, 121.56, 51.45, 34.56, 34.20, 30.25, 28.42; HRMS m/z: 376.1435 (M+1)

N-(1,5-Dihydrazinyl-1,5-dioxopentan-2-yl)-4-(4-oxo-3,4-dihydroquinazolin-2-yl) butanamide (18):

Yield 74.05%, white solid, R_f^a = 0.12, R_f^b = 0.25, m.p. 184-185 °C, IR KBr (cm^{-1}): 3365, 3258, 1750, 1630; ^1H NMR (DMSO-d_6, 400 MHz) δ ppm: 12.13 (s, 1H, Het-NH), 9.11 (s, 1H, NH-hydrazide), 8.90 (s, 1H, NH-hydrazide), 8.05-8.04 (d, 1H, $^{\alpha}$NH-Glu), 7.98-7.40 (m, 4H, Ar-H), 4.16-4.14 (q, 1H, $^{\alpha}$CH), 4.12 (s, 4H, NH_2), 2.59-2.55 (t, 2H, CH_2), 2.19-2.15 (t, 2H, CH_2-Glu), 2.03-1.88 (t, 2H, CH_2), 1.86-1.82 (m, 2H, CH_2), 1.81-1.64 (m, 2H, CH_2-Glu); ^{13}C NMR (DMSO-d_6, 100 MHz) δ ppm: 172.05, 171.40, 171.05, 162.22, 157.48, 149.32, 134.66, 127.26, 126.37, 126.10, 121.31, 51.40, 34.57, 34.22, 30.44, 28.72, 23.16; HRMS m/z: 390.1396 (M+1)

General procedure for the synthesis of hydrazones (5-14 and 19-28)

Compounds **3, 4, 17** and **18** (1 mmol) was dissolved separately in ethanol (10 mL/g of compound) and treated with appropriate aldehydes (2 mmol). The reaction mixture was refluxed for 7–8 h on an oil bath and the completion of reaction was monitored by TLC. After completion of the reaction, solvent was removed under reduced pressure and cooled by adding ice cold water. The resulting precipitate was filtered, washed with water and recrystallized from ethanol to obtain the desired hydrazones (**5-14** and **19-28**).

N-(1,4-bis(2-benzylidenehydrazinyl)-1,4-dioxobutan-2-yl)-3-(4-oxo-3,4-dihydroquinazolin-2-yl)propanamide (5):

Yield 95.26%, white solid, R_f^a = 0.62, R_f^b = 0.70, m.p. 198-200 °C, IR KBr (cm^{-1}): 3250, 3125, 1630, 1615; ^1H NMR (DMSO-d_6, 400 MHz) δ ppm: 12.08 (s, 1H, Het-NH),11.42-11.32 (s, 2H, NH), 8.24-8.04 (s, 2H, CH), 8.22-8.02 (d, 1H, $^{\alpha}$NH-Asp), 7.92-7.36 (m, 14H, Ar-H), 5.64-4.77 (q, 1H, $^{\alpha}$CH), 3.05-3.08 (t, 2H, CH_2), 2.56-2.46 (m, 2H, CH_2-Asp), 2.19-2.22 (t, 2H, CH_2); ^{13}C NMR (DMSO-d_6, 100 MHz) δ ppm: 172.20, 171.45, 162.85, 161.52, 157.2, 149.55, 147.78, 143.56, 134.75, 130.88, 130.18, 129.56, 128.75, 127.87, 127.23, 127.10, 126.32, 126.09, 121.45, 48.90, 35.25, 34.25, 23.42; HRMS m/z: 538.2832 (M+1)

N-(1,4-Dioxo-1,4-bis(2-(3,4,5-trimethoxybenzylidene)hydrazinyl)butan-2-yl)-3-(4-oxo-3,4-dihydroquinazolin-2-yl)propanamide (6):

Yield 93.24%, white solid, R_f^a = 0.59, R_f^b = 0.67, m.p. 192-194 °C, IR KBr (cm^{-1}): 3225, 3115, 1655, 1625; ^1H NMR (DMSO-d_6, 400 MHz) δ ppm: 12.12 (s, 1H, Het-NH),11.44-11.36 (s, 2H, NH), 8.62-8.30 (s, 2H, CH), 8.08-8.06 (d, 1H, $^{\alpha}$NH-Asp), 8.00-6.92 (m, 8H, Ar-H), 5.64-4.77 (q, 1H, $^{\alpha}$CH), 3.77 (s, 18H, OCH_3), 3.65-3.33 (t, 2H, CH_2), 2.99-2.82 (m, 2H, CH_2-Asp), 2.62-2.47 (t, 2H, CH_2); ^{13}C NMR (DMSO-d_6, 100 MHz) δ ppm: 172.62, 171.64, 162.06, 153.58, 149.15, 143.32, 139.45, 139.19, 134.51, 130.14, 127.21, 126.31, 126.08, 121.29, 106.08, 104.63, 60.53, 56.37, 31.69, 30.07; HRMS m/z: 718.1729 (M+1)

N-(1,4-Dioxo-1,4-bis(2-(3,4,5-trihydroxybenzylidene)hydrazinyl)butan-2-yl)-3-(4-oxo-3,4-dihydroquinazolin-2-yl)propanamide (7):

Yield 91.42%, brown solid, R_f^a = 0.44, R_f^b = 0.59, m.p. 184-186 °C, IR KBr (cm^{-1}): 3245, 3200, 1660, 1630; ^1H NMR (DMSO-d_6, 400 MHz) δ ppm: 12.13 (s, 1H, Het-NH),11.30-11.24 (s, 2H, NH), 8.50-8.30 (s, 2H, CH), 8.08-8.05 (d, 1H, $^{\alpha}$NH-Asp), 9.04-7.40 (m, 8H, Ar-H), 6.79-6.55 (s, 6H, OH), 4.77-4.43 (m, 1H, $^{\alpha}$CH), 3.02 (t, 2H, CH_2), 2.58-2.60 (m, 2H, CH_2-Asp), 2.17 (t, 2H, CH_2); ^{13}C NMR (DMSO-d_6, 100 MHz) δ ppm: 162.32, 161.84, 149.98, 146.42, 146.25, 146.15, 136.12, 134.45, 127.98, 126.34, 126.56, 124.65, 121.78, 108.42, 107.35, 106.56, 47.65, 34.87, 34.19, 34.00; HRMS m/z: 634.2342 (M+1)

N-(1,4-*bis*(2-(2,4-Dichlorobenzylidene)hydrazinyl)-1,4-dioxobutan-2-yl)-3-(4-oxo-3,4-dihydroquinazolin-2-yl)propanamide (8):

Yield 92.20%, pale yellow solid, R_f^a = 0.69, R_f^b = 0.79, m.p. 196-198 °C, IR KBr (cm^{-1}): 3295, 3150, 1670, 1635; ^1H NMR (DMSO-d_6, 400 MHz) δ ppm: 12.07 (s, 1H, Het-NH),11.79-11.55 (s, 2H, NH), 8.59-8.27 (s, 2H, CH), 8.25-8.23 (d, 1H, $^{\alpha}$NH-Asp), 8.03-7.26 (m, 10H, Ar-H), 5.64-4.74 (q, 1H, $^{\alpha}$CH), 3.14-2.88 (t, 2H, CH_2), 2.59-2.47 (m, 2H, CH_2-Asp), 2.20-2.16 (t, 2H, CH_2); ^{13}C NMR (DMSO-d_6, 100 MHz) δ ppm: 172.20, 171.28, 162.65, 157.78, 149.98, 138.79, 135.10,

134.20, 133.40, 130.65, 129.28, 128.50, 127.55, 126.98, 126.26, 121.56, 48.50, 34.70, 34.18, 23.25; HRMS m/z: 673.9253 (M+), 675.9218 (M+3), 677.9126 (M+5)

N-(1,4-*bis*(2-(2,4-Dinitrobenzylidene)hydrazinyl)-1,4-dioxobutan-2-yl)-3-(4-oxo-3,4-dihydro quinazolin-2-yl)propanamide (9):

Yield 91.20%, pale pink solid, R_f^a = 0.65, R_f^b = 0.77, m.p. 187-189 °C, IR KBr (cm^{-1}): 3285, 3165, 1665, 1645; ^{1}H NMR (DMSO-d_6, 400 MHz) δ ppm: 12.12 (s, 1H, Het-NH),11.91 (s, 2H, NH), 8.60-8.36 (s, 2H, CH), 8.14 (d, 1H, $^{\alpha}$NH-Asp), 8.98-7.27 (m, 10H, Ar-H), 5.64-4.79 (q, 1H, $^{\alpha}$CH), 3.30-3.04 (t, 2H, CH_2), 2.91-2.67 (m, 2H, CH_2-Asp), 2.60-2.47 (t, 2H, CH_2); ^{13}C NMR (DMSO-d_6, 100 MHz) δ ppm: 171.68, 171.00, 162.05, 161.82, 157.89, 156.98, 149.15, 148.85, 147.96, 134.75, 133.39, 131.67, 129.71, 129.20, 128.07, 127.83, 127.18, 126.06, 125.81, 121.30, 120.99, 120.70, 120.53, 31.66, 30.90, 29.98, 29.49; HRMS m/z: 718.0555 (M+1)

N-(1,4-*bis*(2-Benzylidenehydrazinyl)-1,4-dioxobutan-2-yl)-4-(4-oxo-3,4-dihydroquinazolin-2-yl)butanamide (10):

Yield 90.24%, white solid, R_f^a = 0.60, R_f^b = 0.70, m.p. 219-221 °C, IR KBr (cm^{-1}): 3250, 3145, 1645, 1620; ^{1}H NMR (DMSO-d_6, 400 MHz) δ ppm: 12.10 (s, 1H, Het-NH),11.46-11.34 (s, 2H, NH), 8.24-8.04 (s, 2H, CH), 8.24-8.04 (d, 1H, $^{\alpha}$NH-Asp), 7.94-7.37 (m, 14H, Ar-H), 5.64-4.77 (m, 1H, $^{\alpha}$CH), 3.03-2.95 (t, 2H, CH_2), 2.58-2.47 (m, 2H, CH_2-Asp), 2.19-2.18 (t, 2H, CH_2), 1.94-1.86 (m, 2H, CH_2); ^{13}C NMR (DMSO-d_6, 100 MHz) δ ppm: 172.16, 171.70, 162.19, 161.82, 157.44, 149.33, 147.56, 143.44, 134.63, 130.40, 130.08, 129.20, 128.78, 127.41, 127.24, 127.15, 126.33, 126.10, 121.32, 48.88, 35.00, 34.66, 23.35, 19.30; HRMS m/z: 552.1691 (M+1)

N-(1,4-Dioxo-1,4-bis(2-(3,4,5-trimethoxybenzylidene)hydrazinyl)butan-2-yl)-4-(4-oxo-3,4-dihydroquinazolin-2-yl)butanamide (11):

Yield 94.20%, white solid, R_f^a = 0.56, R_f^b = 0.68, m.p. 212-214 °C, IR KBr (cm^{-1}): 3290, 3175, 1670, 1630; ^{1}H NMR (DMSO-d_6, 400 MHz) δ ppm: 12.14 (s, 1H, Het-NH),11.42-11.34 (s, 2H, NH), 8.60-8.32 (s, 2H, CH), 8.09-8.07 (d, 1H, $^{\alpha}$NH-Asp), 8.02-6.90 (m, 8H, Ar-H), 5.66-4.75 (q, 1H, $^{\alpha}$CH), 3.75 (s, 18H, OCH_3), 3.60-3.35 (t, 2H, CH_2), 2.98-2.72 (m, 2H, CH_2-Asp), 2.68-2.50 (m, 2H, CH_2), 1.94-1.86 (m, 2H, CH_2); ^{13}C NMR (DMSO-d_6, 100 MHz) δ ppm: 172.82, 171.45, 162.25, 153.86, 149.78, 143.65, 139.40, 139.19, 134.98, 130.23, 127.45, 126.28, 126.86, 121.98, 106.45, 104.23, 60.45, 56.78, 31.26, 30.45, 22.65; HRMS m/z: 732.3628 (M+1)

N-(1,4-Dioxo-1,4-bis(2-(3,4,5-trihydroxybenzylidene)hydrazinyl)butan-2-yl)-4-(4-oxo-3,4-dihydroquinazolin-2-yl)butanamide (12):

Yield 91.28%, brown solid, R_f^a = 0.49, R_f^b = 0.57, m.p. 208-210 °C, IR KBr (cm^{-1}): 3290, 3175, 1670, 1630; ^{1}H NMR (DMSO-d_6, 400 MHz) δ ppm: 12.14 (s, 1H, Het-NH),11.31-11.25 (s, 2H, NH), 8.51-8.32 (s, 2H, CH), 8.05-8.04 (d, 1H, $^{\alpha}$NH-Asp), 9.02-7.42 (m, 8H, Ar-H), 6.78-6.56 (s,

6H, OH), 4.78-4.42 (q, 1H, $^{\alpha}$CH), 3.04-3.01 (t, 2H, CH_2), 2.60-2.58 (m, 2H, CH_2-Asp), 2.17-2.14 (t, 2H, CH_2), 1.94-1.92 (m, 2H, CH_2); ^{13}C NMR (DMSO-d_6, 100 MHz) δ ppm: 162.22, 161.08, 149.29, 146.62, 146.55, 146.50, 137.16, 134.69, 127.25, 126.42, 126.15, 124.89, 121.28, 108.10, 107.96, 106.70, 47.25, 34.43, 34.15, 34.00, 22.80; HRMS m/z: 648.1110 (M+1)

N-(1,4-*bis*(2-(2,4-Dichlorobenzylidene)hydrazinyl)-1,4-dioxobutan-2-yl)-4-(4-oxo-3,4-dihydroquinazolin-2-yl)butanamide (13):

Yield 91.02%, pale yellow solid, R_f^a = 0.67, R_f^b = 0.77, m.p. 216-218 °C, IR KBr (cm^{-1}): 3275, 3190, 1665, 1615; ^{1}H NMR (DMSO-d_6, 400 MHz) δ ppm: 12.07 (s, 1H, Het-NH),11.79-11.55 (s, 2H, NH), 8.59-8.27 (s, 2H, CH), 8.25-8.23 (d, 1H, $^{\alpha}$NH-Asp), 8.03-7.26 (m, 10H, Ar-H), 5.64-4.74 (m, 1H, $^{\alpha}$CH), 3.14-2.88 (m, 2H, CH_2), 2.59-2.47 (m, 2H, CH_2-Asp), 2.20-2.16 (t, 2H, CH_2), 1.94-1.92 (m, 2H, CH_2); ^{13}C NMR (DMSO-d_6, 100 MHz) δ ppm: 172.16, 171.86, 162.16, 157.36, 149.31, 138.47, 135.15, 134.56, 133.96, 130.98, 129.70, 128.32, 127.23, 126.30, 126.08, 121.30, 34.77, 34.17, 23.27, 23.13; HRMS m/z: 687.9816 (M+1), 689.9811 (M+3), 691.9719 (M+5)

N-(1,4-*bis*(2-(2,4-Dinitrobenzylidene)hydrazinyl)-1,4-dioxobutan-2-yl)-4-(4-oxo-3,4-dihydroquinazolin-2-yl)butanamide (14):

Yield 86.75%, pale pink solid, R_f^a = 0.65, R_f^b = 0.72, m.p. 206-208 °C, IR KBr (cm^{-1}): 3275, 3175, 1672, 1612; ^{1}H NMR (DMSO-d_6, 400 MHz) δ ppm: 12.14 (s, 1H, Het-NH),11.93 (s, 2H, NH), 8.58-8.37 (s, 2H, CH), 8.11-8.12 (d, 1H, $^{\alpha}$NH-Asp), 8.90-7.25 (m, 10H, Ar-H), 5.62-4.80 (q, 1H, $^{\alpha}$CH), 3.28-3.07 (t, 2H, CH_2), 2.92-2.64 (m, 2H, CH_2-Asp), 2.64-2.44 (t, 2H, CH_2), 2.01-1.98 (m, 2H, CH_2); ^{13}C NMR (DMSO-d_6, 100 MHz) δ ppm: 171.85, 171.21, 162.02, 161.65, 157.80, 156.86, 156.32, 149.19, 148.20, 147.99, 134.65, 133.40, 131.13, 129.77, 128.56, 128.18, 127.96, 126.35, 125.90, 121.26, 120.18, 120.16, 31.70, 30.80, 29.58, 29.51, 22.65; HRMS m/z: 732.1242 (M+1)

N-(1,5-*bis*(2-Benzylidenehydrazinyl)-1,5-dioxopentan-2-yl)-3-(4-oxo-3,4-dihydroquinazolin-2-yl)propanamide (19):

Yield 89.22%, white solid, R_f^a = 0.62, R_f^b = 0.75, m.p. 230-232 °C, IR KBr (cm^{-1}): 3258, 3130, 1675, 1618; ^{1}H NMR (DMSO-d_6, 400 MHz) δ ppm: 12.14 (s, 1H, Het-NH),11.48-11.33 (s, 2H, NH), 8.20-8.02 (s, 2H, CH), 8.20-8.02 (d, 1H, $^{\alpha}$NH-Glu), 7.92-7.38 (m, 14H, Ar-H), 5.66-4.76 (q, 1H, $^{\alpha}$CH), 3.05-3.02 (t, 2H, CH_2), 2.54-2.43 (t, 2H, CH_2-Glu), 2.19-2.15 (t, 2H, CH_2), 2.02-1.92 (m, 2H, CH_2-Glu); ^{13}C NMR (DMSO-d_6, 100 MHz) δ ppm: 172.20, 171.60, 162.56, 161.80, 157.40, 149.32, 147.86, 143.20, 134.95, 130.45, 130.10, 129.56, 128.70, 127.75, 127.63, 127.10, 126.85, 126.09, 121.30, 48.80, 35.12, 34.09, 28.09, 23.38; HRMS m/z: 552.2365 (M+1)

N-(1,5-Dioxo-1,5-bis(2-(3,4,5-trimethoxybenzylidene)hydrazinyl)pentan-2-yl)-3-(4-oxo-3,4-dihydroquinazolin-2-yl)propanamide (20):

Yield 90.50%, white solid, R_f^a = 0.61, R_f^b = 0.72, m.p. 244-247 °C, IR KBr (cm^{-1}): 3258, 3130, 1675, 1618; ^1H NMR (DMSO-d_6, 400 MHz) δ ppm: 12.13 (s, 1H, Het-NH),11.42-11.33 (s, 2H, NH), 8.60-8.29 (s, 2H, CH), 8.09-8.05 (d, 1H, $^{\alpha}$NH-Glu), 8.01-6.95 (m, 8H, Ar-H), 5.65-4.45 (q, 1H, $^{\alpha}$CH), 3.78 (s, 18H, OCH_3), 3.62-3.35 (t, 2H, CH_2), 2.98-2.81 (t, 2H, CH_2-Glu), 2.62-2.45 (t, 2H, CH_2), 2.09-1.95 (m, 2H, CH_2-Glu); ^{13}C NMR (DMSO-d_6, 100 MHz) δ ppm: 172.78, 171.34, 162.04, 153.62, 149.29, 143.38, 139.44, 139.23, 134.65, 130.25, 127.26, 126.38, 126.26, 121.45, 106.26, 104.56, 60.56, 56.89, 31.75, 30.28, 28.42; HRMS m/z: 732.2498 (M+1)

N-(1,5-Dioxo-1,5-bis(2-(3,4,5-trihydroxybenzylidene)hydrazinyl)pentan-2-yl)-3-(4-oxo-3,4-dihydroquinazolin-2-yl)propanamide (21):

Yield 93.18%, brown solid, R_f^a = 0.45, R_f^b = 0.59, m.p. 234-238 °C, IR KBr (cm^{-1}): 3235, 3115, 1660, 1612; ^1H NMR (DMSO-d_6, 400 MHz) δ ppm: 12.12 (s, 1H, Het-NH),11.14-11.05 (s, 2H, NH), 8.90-8.78 (s, 2H, CH), 8.90-8.78 (d, 1H, $^{\alpha}$NH-Glu), 8.28-7.40 (m, 8H, Ar-H), 6.59 (s, 6H, OH), 4.30-5.21 (q, 1H, $^{\alpha}$CH), 2.83-2.78 (t, 2H, CH_2), 2.69-2.64 (t, 2H, CH_2-Glu), 2.21-2.15 (t, 2H, CH_2), 1.95-1.89 (m, 2H, CH_2-Glu); ^{13}C NMR (DMSO-d_6, 100 MHz) δ ppm:173.53, 171.68, 171.55, 167.80, 162.11, 157.09, 149.18, 148.41, 147.14, 146.57, 144.32, 136.19, 135.90, 134.63, 127.20, 126.35, 126.10, 125.03, 124.91, 121.29, 106.75, 106.65, 106.45, 31.71, 31.01, 30.27, 30.13, 28.34; HRMS m/z: 648.1110 (M+1)

N-(1,5-*bis*(2-(2,4-Dichlorobenzylidene)hydrazinyl)-1,5-dioxopentan-2-yl)-3-(4-oxo-3,4-dihydroquinazolin-2-yl)propanamide (22):

Yield 90.80%, pale yellow, solid, R_f^a = 0.67, R_f^b = 0.79, m.p. 230-232 °C, IR KBr (cm^{-1}): 3240, 3125, 1665, 1618; ^1H NMR (DMSO-d_6, 400 MHz) δ ppm: 12.12 (s, 1H, Het-NH),11.79-11.40 (s, 2H, NH), 8.53-8.31 (s, 2H, CH), 8.53-8.31 (s, 1H, $^{\alpha}$NH-Glu), 8.25-7.21 (m, 10H, Ar-H), 5.22-4.33 (m, 1H, $^{\alpha}$CH), 2.81 (m, 2H, CH_2), 2.69 (brd s, 2H, CH_2-Glu), 2.28 (t, 2H, CH_2), 2.01-1.93 (m, 2H, CH_2-Glu); ^{13}C NMR (DMSO-d_6, 100 MHz) δ ppm: 174.14, 173.67, 171.746, 168.46, 162.00, 156.98, 149.16, 142.40, 138.71, 137.90, 135.51, 134.97, 133.85, 131.52, 130.936, 129.645, 128.71, 127.14, 126.26, 121.31, 52.01, 49.00, 31.53, 30.06, 28.73, 26.77; HRMS m/z: 687.9931 (M+1), 689.9811 (M+3), 691.9835 (M+5)

N-(1,5-*bis*(2-(2,4-Dinitrobenzylidene)hydrazinyl)-1,5-dioxopentan-2-yl)-3-(4-oxo-3,4-dihydroquinazolin-2-yl)propanamide (23):

Yield 91.42%, pale pink solid, R_f^a = 0.56, R_f^b = 0.69, m.p. 218-220 °C, IR KBr (cm^{-1}): 3286, 3156, 1675, 1630; ^1H NMR (DMSO-d_6, 400 MHz) δ ppm: 12.04 (s, 1H, Het-NH),11.90-11.72 (s, 2H, NH), 8.20-8.15 (s, 2H, CH), 8.05 (s, 1H, $^{\alpha}$NH-Glu), 8.68-7.30 (m, 10H, Ar-H), 5.21-4.35 (q, 1H, $^{\alpha}$CH), 2.75-2.72 (t, 2H, CH_2), 2.63-2.60 (t, 2H, CH_2-Glu), 2.48-2.36 (t, 2H, CH_2), 2.20-2.15 (m, 2H, CH_2-Glu); ^{13}C NMR (DMSO-d_6, 100 MHz) δ ppm: 174.95, 172. 56, 162.09, 157.65, 149.78, 147.35, 147.60, 134.86, 129.78, 128.74, 127.45, 127.42, 126.78, 126.70, 121.50, 120.45, 34.76, 34.86, 28.68, 27.58; HRMS m/z: 732.3248 (M+1)

N-(1,5-*bis*(2-Benzylidenehydrazinyl)-1,5-dioxopentan-2-yl)-4-(4-oxo-3,4-dihydroquinazolin-2-yl)butanamide (24):

Yield 90.12%, white solid, $R_f^a = 0.62$, $R_f^b = 0.72$, m.p. 202-204 °C, IR KBr (cm^{-1}): 3272, 3146, 1672, 1625; ^1H NMR (DMSO-d_6, 400 MHz) δ ppm: 12.12 (s, 1H, Het-NH),11.50-11.32 (s, 2H, NH), 8.18-8.04 (s, 2H, CH), 8.18-8.02 (d, 1H, $^{\alpha}$NH-Glu), 7.90-7.36 (m, 14H, Ar-H), 5.64-4.75 (q, 1H, $^{\alpha}$CH), 3.06-3.02 (t, 2H, CH_2), 2.55-2.40 (t, 2H, CH_2-Glu), 2.14-2.11 (t, 2H, CH_2), 2.01-1.91 (m, 2H, CH_2-Glu), 1.90-1.82 (m, 2H, CH_2); ^{13}C NMR (DMSO-d_6, 100 MHz) δ ppm: 172.40, 171.82, 162.92, 161.24, 157.32, 149.65, 147.28, 143.85, 134.78, 130.20, 130.14, 129.78, 128.65, 127.50, 127.38, 127.09, 126.29, 126.12, 121.23, 48.56, 46.22, 35.15, 34.08, 28.10, 23.32; HRMS m/z: 566.3023 (M+1)

N-(1,5-Dioxo-1,5-bis(2-(3,4,5-trimethoxybenzylidene)hydrazinyl)pentan-2-yl)-4-(4-oxo-3,4-dihydroquinazolin-2-yl)butanamide (25):

Yield 89.24%, white solid, $R_f^a = 0.61$, $R_f^b = 0.70$, m.p. 204-206 °C, IR KBr (cm^{-1}): 3265, 3140, 1675, 1632; ^1H NMR (DMSO-d_6, 400 MHz) δ ppm: 12.11 (s, 1H, Het-NH),11.52-11.13 (s, 2H, NH), 8.22-8.11 (s, 2H, CH), 8.03-8.01 (d, 1H, $^{\alpha}$NH-Glu), 7.95-6.80 (m, 8H, Ar-H), 5.21-4.35 (m, 1H, $^{\alpha}$CH), 3.77 (s, 18H, OCH_3), 2.68 (m, 2H, CH_2), 2.59 (m, 2H, CH_2-Glu), 2.47 (m, 2H, CH_2), 2.32-2.21 (m, 2H, CH_2-Glu), 1.94-1.93 (m, 2H, CH_2); ^{13}C NMR (DMSO-d_6, 100 MHz) δ ppm: 173.94, 168.50, 168.16, 162.19, 157. 46, 157.37, 153.56, 149.32, 139.69, 139.34, 134.59, 130.28, 130.09, 127.23, 126.34, 126.09, 121.33, 104.78, 104.61, 104.40, 60.50, 56.35, 34.76, 34.60, 34.23, 28.90, 23.28, 23.20; HRMS m/z: 746.2114 (M+1)

N-(1,5-Dioxo-1,5-bis(2-(3,4,5-trihydroxybenzylidene)hydrazinyl)pentan-2-yl)-4-(4-oxo-3,4-dihydroquinazolin-2-yl)butanamide (26):

Yield 93.52%, brown solid, $R_f^a = 0.42$, $R_f^b = 0.56$, m.p. 198-200 °C, IR KBr (cm^{-1}): 3255, 3135, 1672, 1622; ^1H NMR (DMSO-d_6, 400 MHz) δ ppm: 12.10 (s, 1H, Het-NH),11.12-11.08 (s, 2H, NH), 8.92-8.76 (s, 2H, CH), 8.92-8.76 (d, 1H, $^{\alpha}$NH-Glu), 8.27-7.41 (m, 8H, Ar-H), 6.58 (s, 6H, OH), 5.20-4.23 (q, 1H, $^{\alpha}$CH), 2.81-2.74 (t, 2H, CH_2), 2.65-2.61 (t, 2H, CH_2-Glu), 2.22-2.18 (t, 2H, CH_2), 1.99-1.95 (m, 2H, CH_2-Glu), 1.90-1.85 (m, 2H, CH_2); ^{13}C NMR (DMSO-d_6, 100 MHz) δ ppm:173.50, 171.16, 171.09, 167.52, 162.10, 157.19, 149.56, 148.95, 147.16, 146.06, 144.30, 136.93, 135.62, 134.55, 127.18, 126.25, 126.75, 125.26, 124.56, 121.30, 106.78, 106.45, 106.25, 31.85, 31.10, 30.18, 30.12, 28.18; HRMS m/z: 662.2619 (M+1)

N-(1,5-*bis*(2-(2,4-Dichlorobenzylidene)hydrazinyl)-1,5-dioxopentan-2-yl)-4-(4-oxo-3,4-dihydroquinazolin-2-yl)butanamide (27):

Yield 90.58%, pale yellow solid, $R_f^a = 0.67$, $R_f^b = 0.78$, m.p. 232-234 °C, IR KBr (cm^{-1}): 3245, 3132, 1670, 1625; ^1H NMR (DMSO-d_6, 400 MHz) δ ppm: 12.14 (s, 1H, Het-NH),11.82-11.45 (s, 2H, NH), 8.55-8.30 (s, 2H, CH), 8.55-8.30 (s, 1H, $^{\alpha}$NH-Glu), 8.23-7.20 (m, 10H, Ar-H), 5.25-

4.30 (q, 1H, $^{\alpha}$CH), 2.85-2.82 (t, 2H, CH_2), 2.67-2.63 (t, 2H, CH_2-Glu), 2.30-2.27 (t, 2H, CH_2), 2.02-1.94 (m, 2H, CH_2-Glu), 1.92-1.89 (m, 2H, CH_2); ^{13}C NMR (DMSO-d_6, 100 MHz) δ ppm: 174.20, 173.68, 171.74, 168.40, 162.05, 156.90, 149.20, 142.28, 138.75, 137.85, 135.55, 134.90, 133.82, 131.45, 130.85, 129.62, 128.75, 128.09, 127.20, 126.30, 121.25, 52.08, 49.05, 31.55, 30.02, 28.78, 26.74; HRMS m/z: 701.9232 (M+1), 703.9180 (M+3), 705.9082 (M+5)

N-(1,5-*bis*(2-(2,4-Dinitrobenzylidene)hydrazinyl)-1,5-dioxopentan-2-yl)-4-(4-oxo-3,4-dihydroquinazolin-2-yl)butanamide (28):

Yield 88.56%, white solid, R_f^a = 0.62, R_f^b = 0.70, m.p. 222-224 °C, IR KBr (cm^{-1}): 3255, 3145, 1663, 1615; ^{1}H NMR (DMSO-d_6, 400 MHz) δ ppm: 12.06 (s, 1H, Het-NH),11.93-11.76 (s, 2H, NH), 8.25-8.20 (s, 2H, CH), 8.04 (s, 1H, $^{\alpha}$NH-Glu), 8.66-7.39 (m, 10H, Ar-H), 5.20-4.37 (q, 1H, $^{\alpha}$CH), 2.74-2.70 (t, 2H, CH_2), 2.64-2.61 (t, 2H, CH_2-Glu), 2.47-2.44 (t, 2H, CH_2), 2.20-2.15 (m, 2H, CH_2-Glu), 1.93-1.90 (m, 2H, CH_2); ^{13}C NMR (DMSO-d_6, 100 MHz) δ ppm: 174.49, 172.27, 162.07, 157.35, 149.21, 147.90, 147.69, 134.56, 129.70, 128.00, 127.56, 127.15, 126.31, 126.01, 121.16, 120.69, 34.68, 34.19, 28.69, 23.22; HRMS m/z: 746.091 (M+1)

Biological activity

Antioxidant activities

DPPH (1,1-diphenyl-2-picryl-hydrazyl) assay

The radical scavenging activity of DPPH free radicals by synthesized compounds was determined according to the reported method.[1] Briefly, 50 µL of test compounds was mixed at different concentrations (25, 50, 100, 200 and 300 µg/mL) with 1 mL of 0.1 mM DPPH in methanol solution and 450 µL of 50 mM Tris HCl buffer (pH 7.4). Methanol (50 µL) only was used as the experimental control. After 30 min of incubation at room temperature, the reduction in the number of DPPH free radicals was measured by reading the absorbance at 517 nm. AA and GA were used as control similar to test concentrations. Percent inhibition was calculated from the following equation:

$$\% \text{ Inhibition} = \left[\frac{\text{Absorbance of control - Absorbance of test sample}}{\text{Absorbance of control}}\right] \times 100$$

DMPD (*N, N*-dimethyl-*p*-phenylenediamine) assay

The DMPD radical scavenging ability of synthesized compounds was determined by the Fogliano *et al.,* method,[2] with slight modifications by Gulcin.[3] This assay is based on the capacity of the extract to inhibit $DMPD^{\cdot +}$ cation radical formation. Briefly, 105 mg of DMPD was dissolved in 5 mL of distilled water. Then, 1 mL of this solution was added to 100 mL of 0.1 M acetate buffer (pH 5.3). $DMPD^{\cdot +}$ was produced by adding 0.3 mL ferric chloride (0.05 M) to this solution. Different concentrations of standard antioxidants or synthesized compounds (25, 50, 100, 200 and 300 µg/mL) were added, and the total volume was adjusted to 1 mL with distilled water. One millilitre of the $DMPD^{\cdot +}$ solution was directly added to the reaction mixture. The reaction mixtures were incubated in the dark for 15 minutes and the absorbance was measured at 505 nm.

$$\textbf{\% Inhibition} = \left[\frac{\textbf{Absorbance of control - Absorbance of test sample}}{\textbf{Absorbance of control}}\right] \times \textbf{100}$$

ABTS (2,2-azinobis-(3-ethylbenzothiazoline-6-sufonic acid) assay

The ability of the test sample to scavenge $ABTS^{\cdot +}$ radical cation was determined according to the literature method with slight modifications.[4] The $ABTS^{\cdot +}$ radical cation was pregenerated by mixing 7 mM $ABTS^{\cdot +}$ stock solutions with 2.45 mM potassium persulfate (final concentration) and incubating for 12–16 hrs in the dark at room temperature until the reaction was complete and the absorbance was stable. The absorbance of the $ABTS^{\cdot +}$ solution was equilibrated to 0.70 (± 0.02) by diluting with distilled water at room temperature, then 2 mL was mixed with different concentration of the test sample (25, 50, 100, 200, and 300 µg/mL) and the absorbance was measured at 734 nm after 6 minutes. The scavenging capability of $ABTS^{\cdot +}$ radical was calculated using the following equation:

$ABTS^{\cdot +}$ scavenging effect (%) = $[(A_c - A_s) / A_c] \times 100$

Where, A_c is the initial concentration of the $ABTS^{\cdot +}$ and A_s is the absorbance of the remaining concentration of $ABTS^{\cdot +}$ in the presence of compounds.

Anti-inflammatory activity

Human erythrocyte suspension

The whole blood was collected from a healthy volunteer who had not taken any NSAIDS for 2 weeks prior to the experiment and collected in heparinized vacutainer. The blood was washed three times with 0.9% saline and centrifuged simultaneously for 10 minutes at 3000 rpm. The packed cells were washed with 0.9% saline and 40% v/v suspension made using isotonic phosphate buffer which was composed of 154 mM NaCl in 10 mM Sodium Phosphate Buffer at pH 7.4 used as Stock erythrocyte or RBC suspension.

Hypotonic solution-induced haemolysis

The membrane stabilizing activity of the compounds was assessed according to the reported method[5] with slight modification. The test sample consisted of stock erythrocyte (RBC) suspension 0.5 mL mixed with 5 mL of hypotonic solution (50 mM NaCl in 10 mM Sodium Phosphate Buffered saline at pH 7.4) containing different concentrations of sample (25, 50, 100, 200 and 300 μg/mL). The control consists of 0.5 mL RBC suspension mixed with 5 mL of hypotonic buffered solution alone. The standard drug acetylsalicylic was treated similar to test concentration. The experiment was carried out in triplicate. The mixtures were incubated for 10 minutes at room temperature, centrifuged for 10 minutes at 3000 rpm and absorbance of the supernatant was measured spectrophotometrically at 540 nm. The percentage inhibition of haemolysis or membrane stabilization was calculated from the following equation.

$$\% \text{ Inhibition of haemolysis} = \left[\frac{A_1 - A_2}{A_1} \right] \times 100$$

Where:

A_1 = Absorbance of hypotonic buffered solution alone

A_2 = Absorbance of test /standard sample in hypotonic solution

Antimicrobial activity

Antibacterial activity

In vitro antibacterial activity was evaluated against human pathogens of both gram negative pathogen *Escherichia coli* and gram positive organism *Staphylococcus aureus* by agar well diffusion method.[6] All the assays were performed in triplicates.

The microorganisms (hospital cultures) were inoculated in to the sterilized nutrient broth and maintained at 37 °C for 24 hrs. On the day of testing, bacteria were subculture separately into 25 mL of sterilized nutrient broth. Inoculated subculture broths were kept at room temperature for the growth of inoculums. Each test compound (**1-28**) and standard drug of 10 mg was dissolved in 10 mL of DMSO to get a concentration of 1 μg/mL and further diluted to get a final concentration of 50 μg/mL. About 15-20 mL of molten nutrient agar was poured into each of the sterile plates. With the help of cork borer of 6 mm diameter, the cups were punched and scooped out of the set agar and the plates were inoculated with the suspension of particular organism by spread plate technique. The cups of inoculated plates were then filled with 0.1 mL of the test solution, streptomycin solution and DMSO (negative control). The plates were allowed to stay for 24 h at 37 °C and zone of inhibition (mm) was then measured.

Antifungal activity

In vitro antifungal activity was evaluated against two fungal species namely *Fusarium moniliforme* and *Aspergillus niger* by agar well diffusion method.[7] The fungal strains were subculture separately into 25 mL of sterilized nutrient broth and compounds and standard drug (bavistin) of 10 mg was dissolved in 10 mL of DMSO to get a concentration of 1 mg/ml and further diluted to get a final concentration of 50 μg/mL. Molten media of sabouraud agar of 10-15 mL was poured into the petri plates and allowed to solidify. Fungal subculture was inoculated on the solidified media. With the help of 6 mm cork borer, the cups were punched and scooped out of the set agar. The cups of inoculated plates were then filled with 0.1 mL of the test solution, bavistin solution and DMSO (negative control). The plates were allowed to stay for 3 days at room temperature and zone of inhibition (mm) was then measured.

Molecular docking studies

Maestro 9.3.5 version of the Schrodinger software suite, 2011 is used to obtained binding interaction of molecules with targeted site. The 3D crystallographic structure of proteins (PDB ID: 2HCK, 1CX2 and 2VF5) was retrieved from Protein Data Bank (www.rcsb.org/pdb). The lowest energy states of ligand were achieved using LigPrep program and it was optimize by force field OPLS-2005 (Optimized Potential for Liquid Simulations). The protein structures were pre-processed, modify and refined by Protein Preparation Wizard. Further, it was minimized by OPLS-2005 force field. The protein and ligand interaction performed by generation of receptor grid in the target site of protein by GLIDE. Depending on the extent of docking the score were produced (docking score) it will determine the best fitted ligand to target protein.

References

1. L. Blois, *Nature*, 1958, 181, 1199–1200.

2. V. Fogliano, V. Verde, G. Randazzo and A. Ritieni, *J. Agric. Food Chem.*, 1999, 47, 1035–1040.

3. I. Glucin, *Innov. food sci. & emerg. Technol.*, 2010, 11, 210–218.

4. R. Re, N. Pellegrini, A. Proteggente, A. Pannala, M. Yang and C. Rice-Evan, *Free. Radic. Biol. Med.*, 1999, 26, 1231–1237.

5. U. A. Shinde, A. S. Phadke, A. M. Nair, A. A. Mungantiwar, V. J. Dikshit and M. N. Saraf, *Fitoterapia*, 1999, 70, 251–257.

6. C. Perez, M. Paul and P. Bazerque, *Acta Biol. Med. Exp.*, 1990, 56, 113–115.

7. I. Singh and V.P. Singh, *Phytomorphology*, 2000, 50, 151–157.

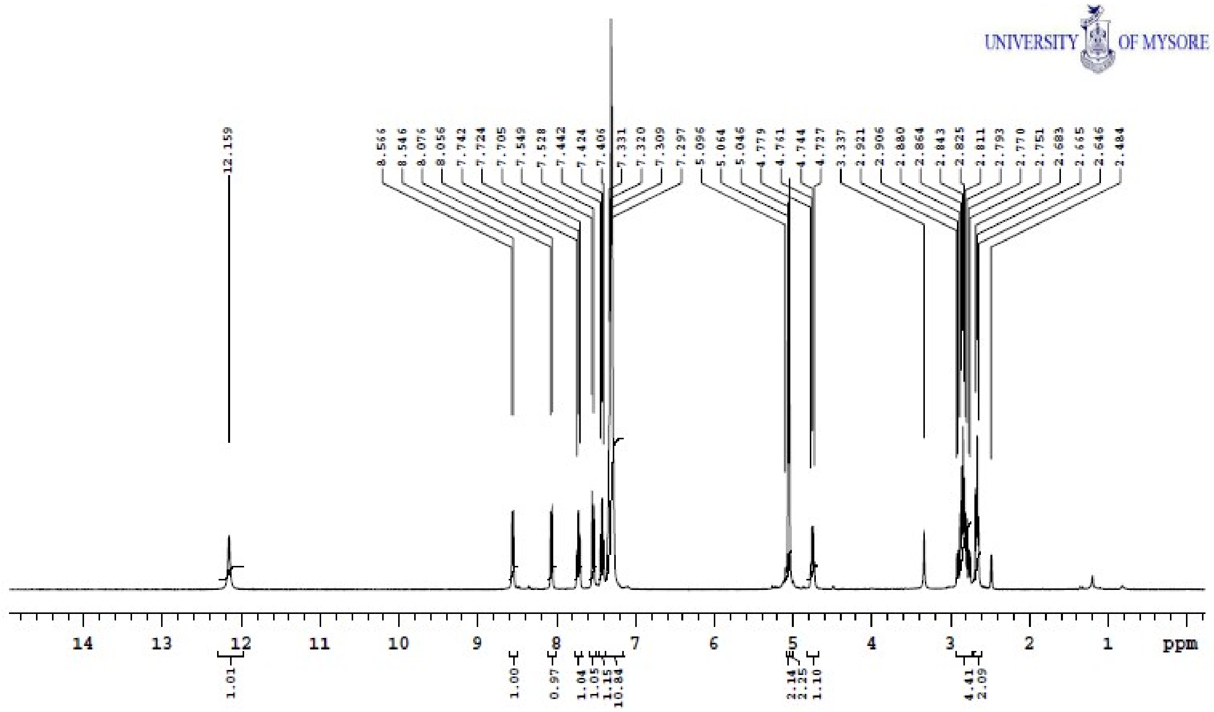

^{1}H NMR spectrum of compound 1

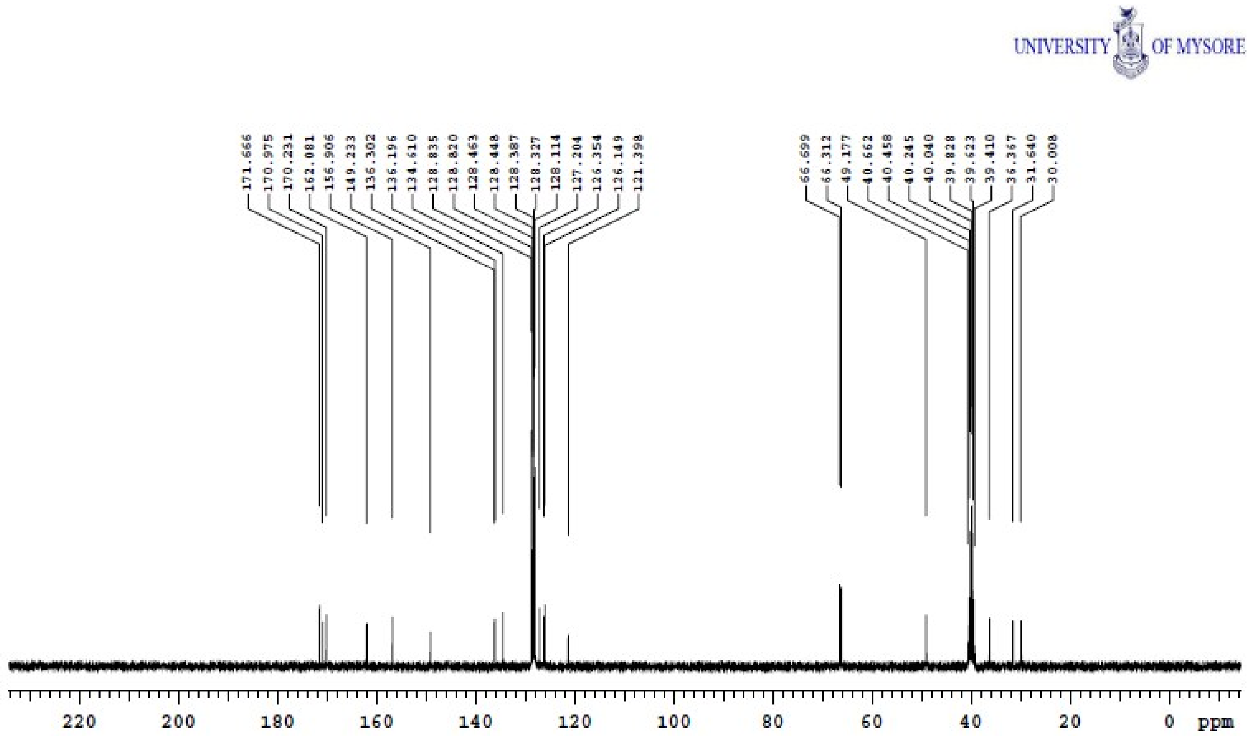

^{13}C NMR spectrum of compound 1

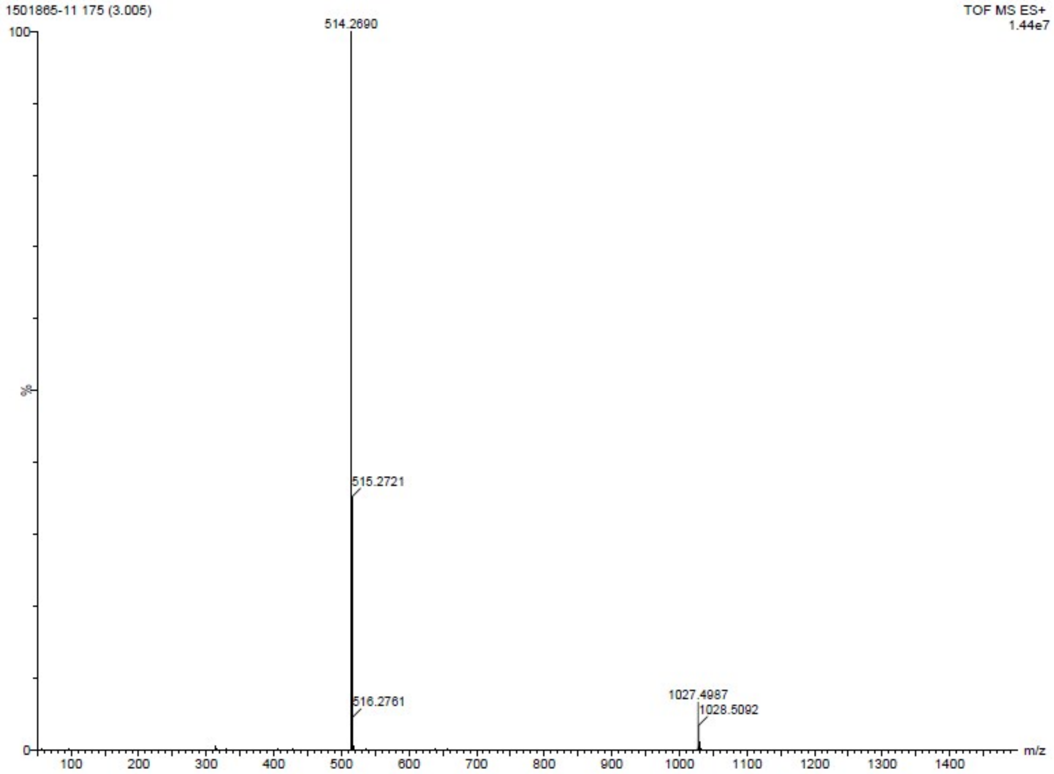

Mass spectrum of compound 1

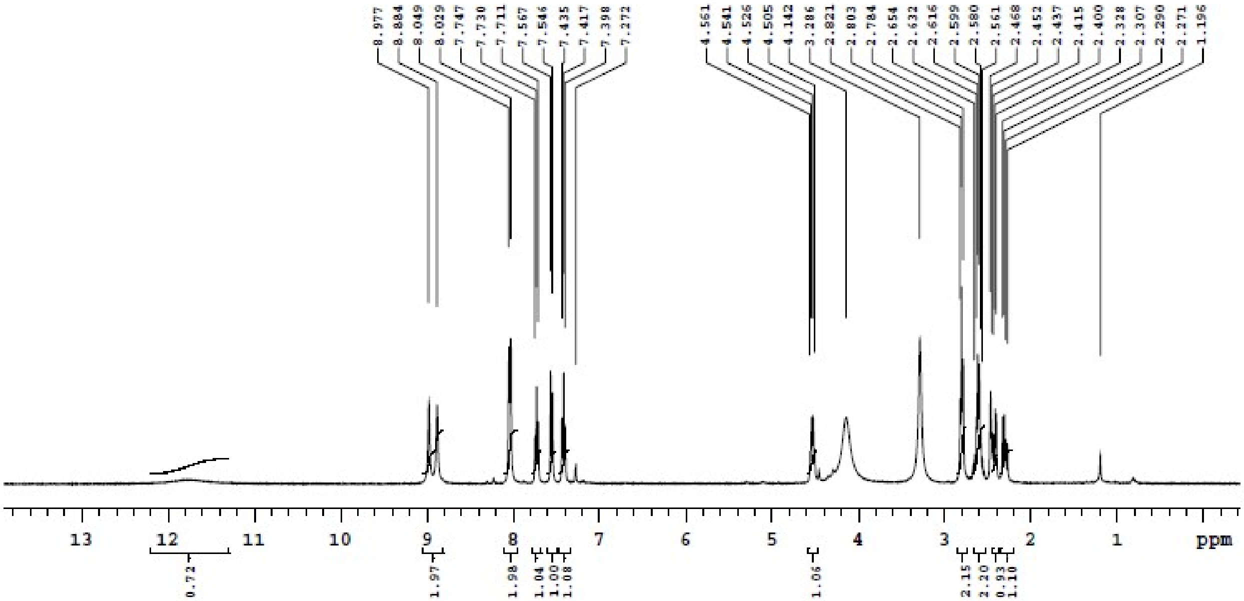

^{1}H NMR spectrum of compound 3

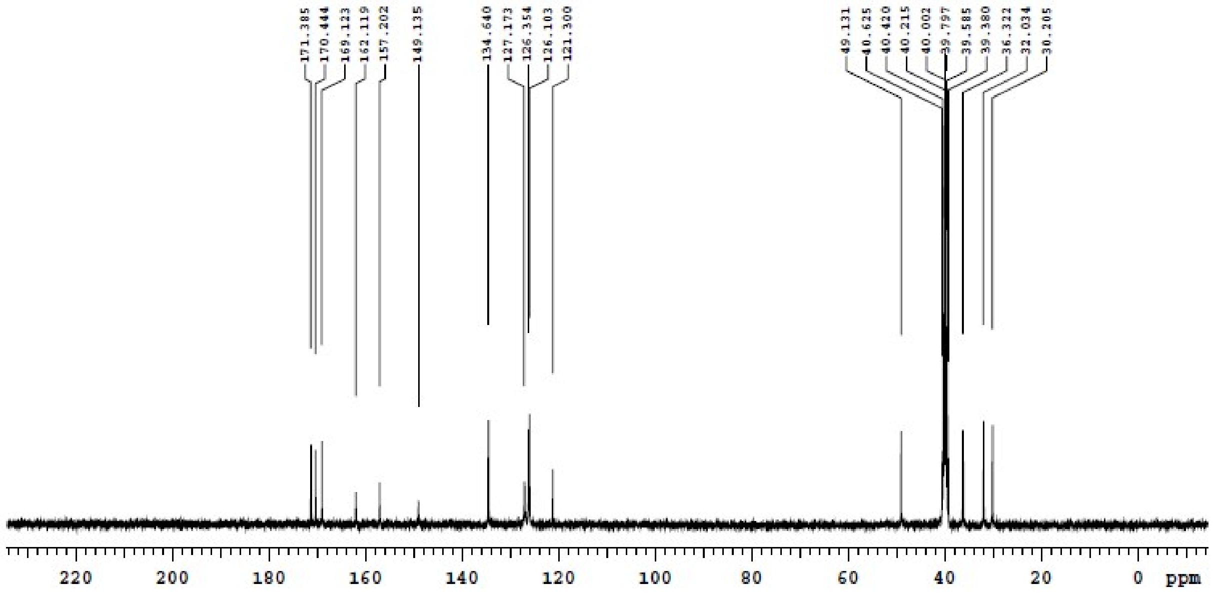

^{13}C NMR spectrum of compound 3

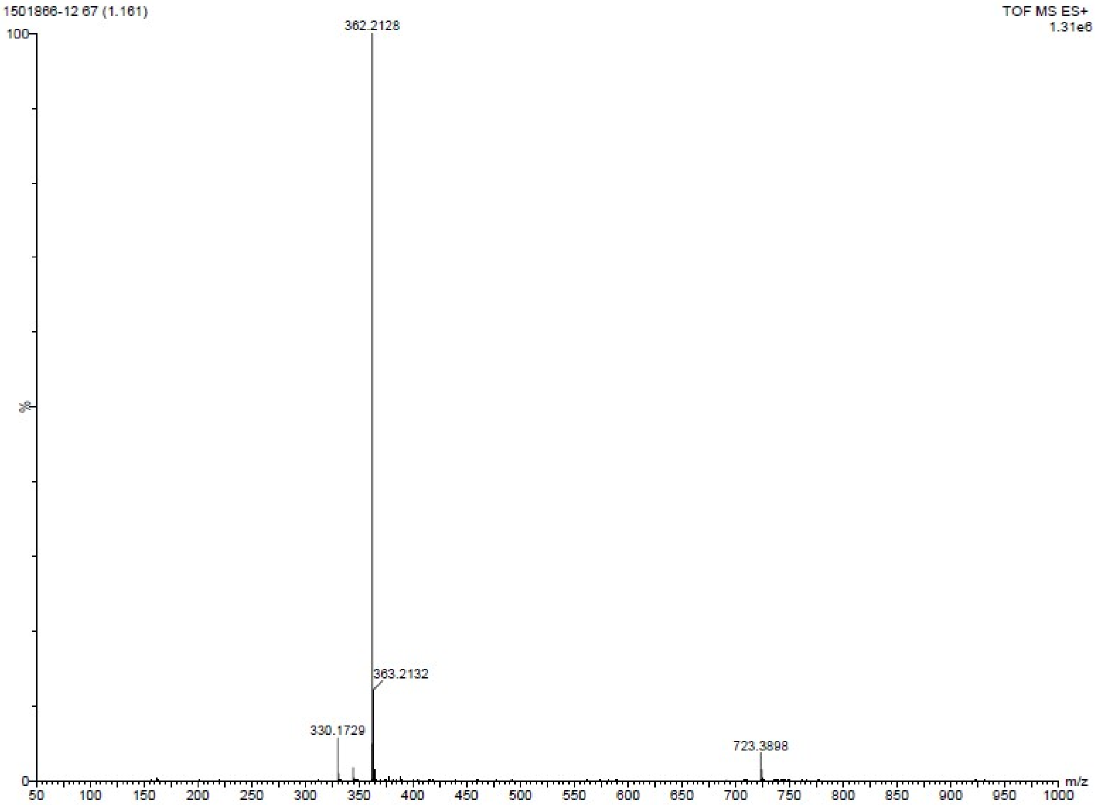

Mass spectrum of compound 3

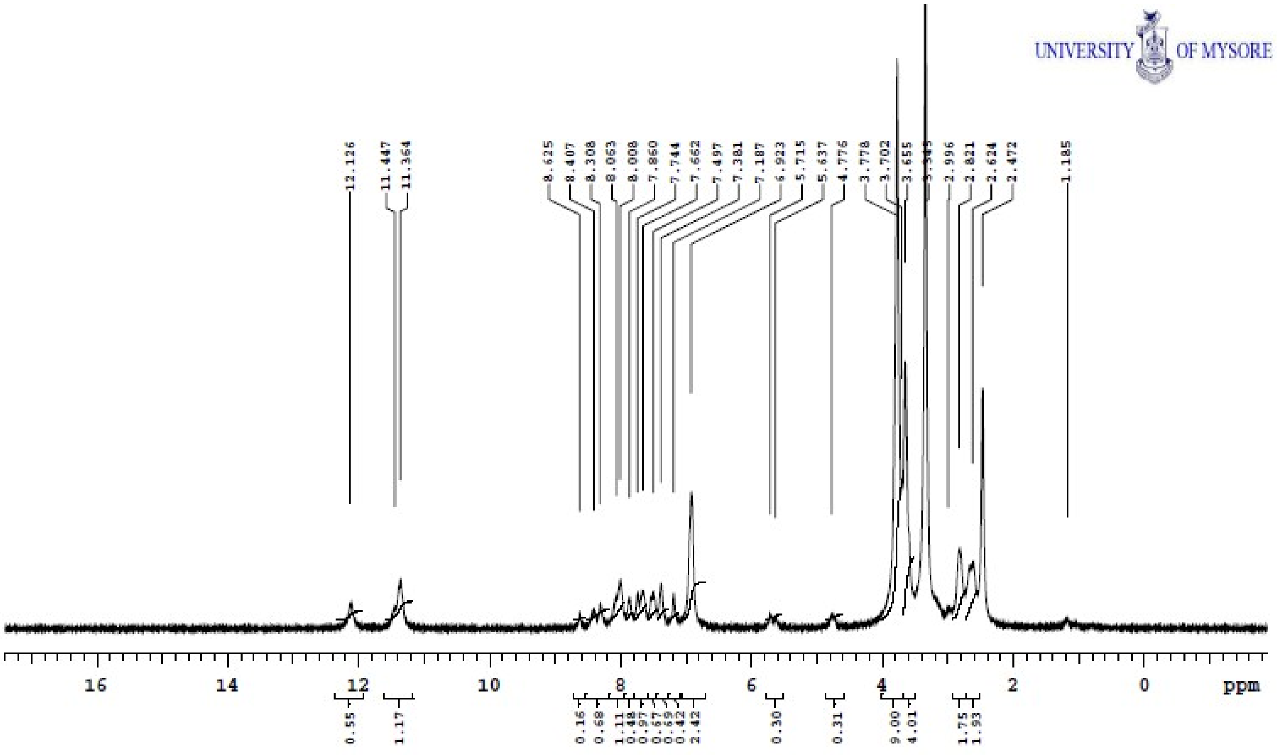

^{1}H NMR spectrum of compound 6

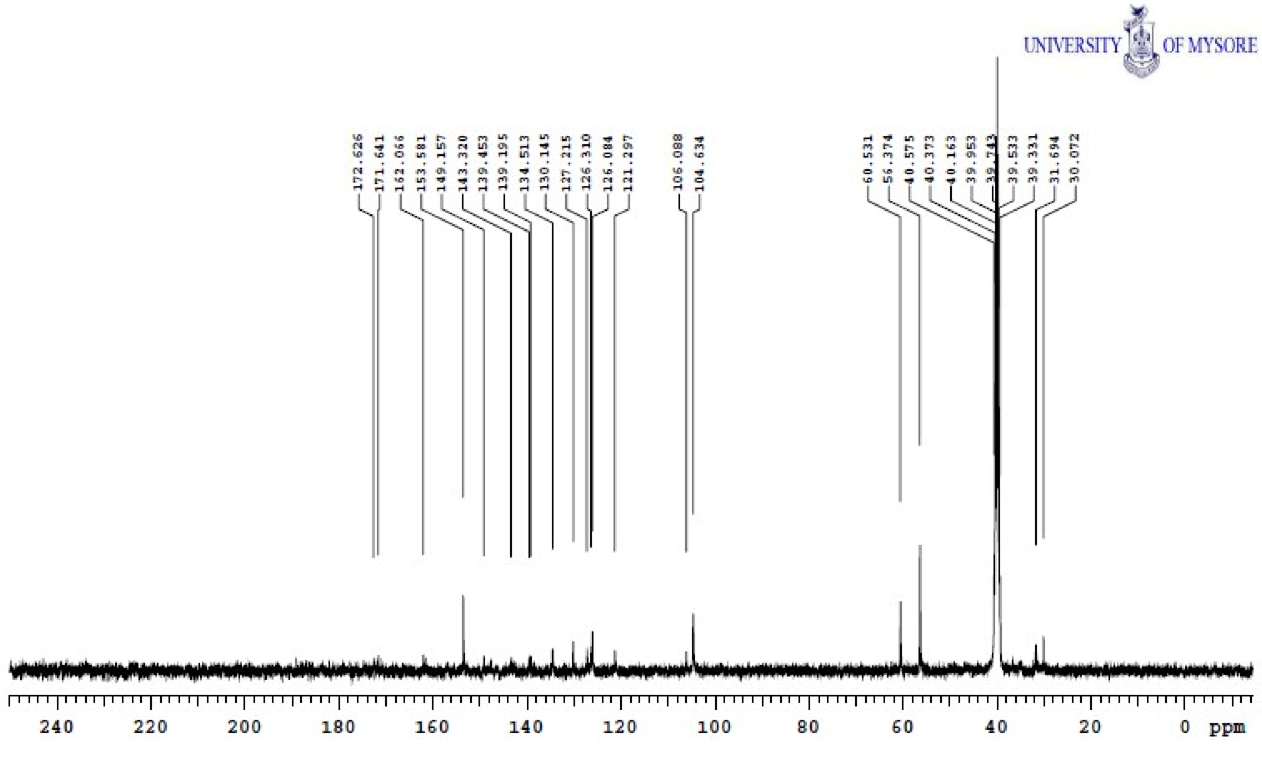

^{13}C NMR spectrum of compound 6

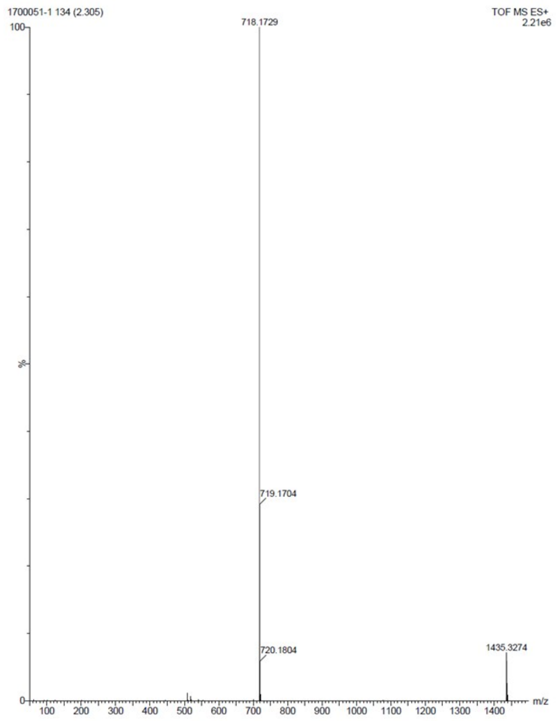

Mass spectrum of compound 6

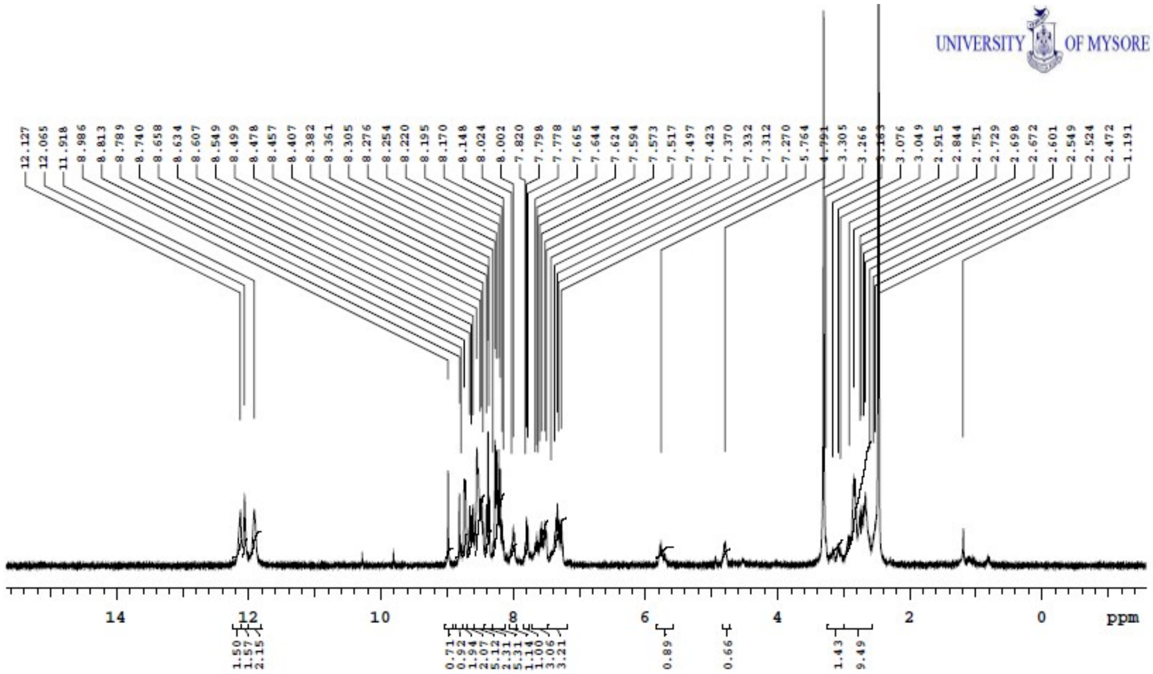

^1H NMR spectrum of compound 9

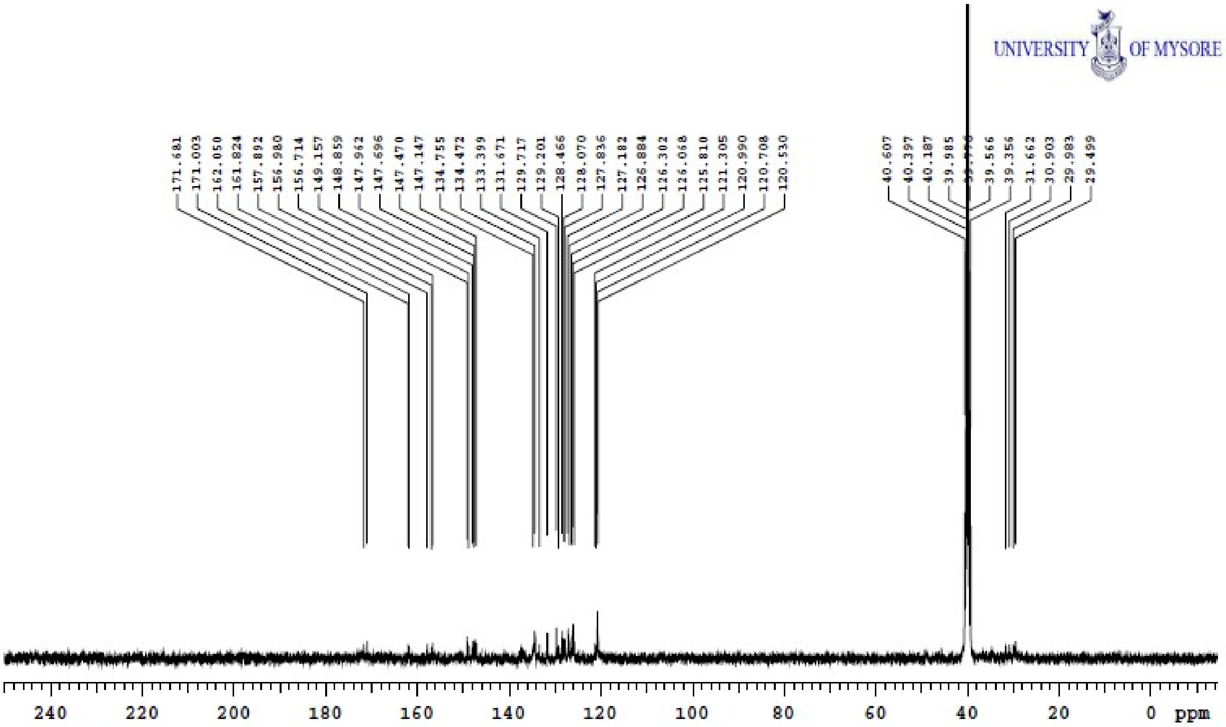

^{13}C NMR spectrum of compound 9

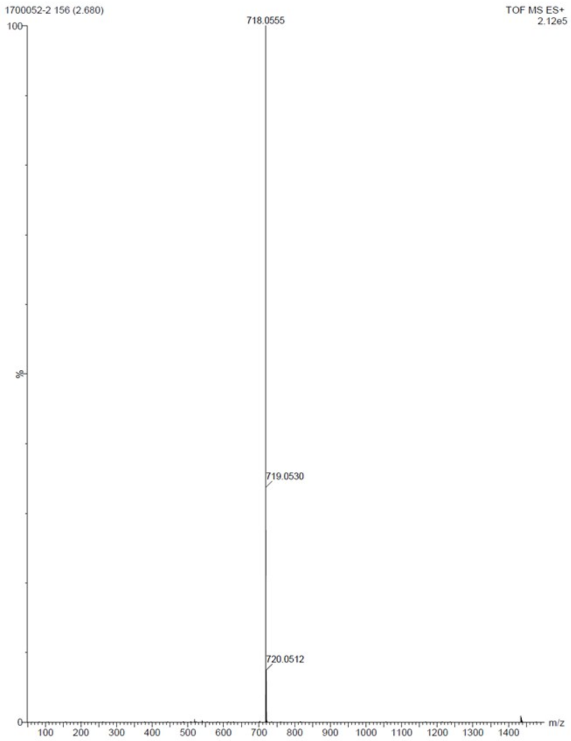

Mass spectrum of compound 9

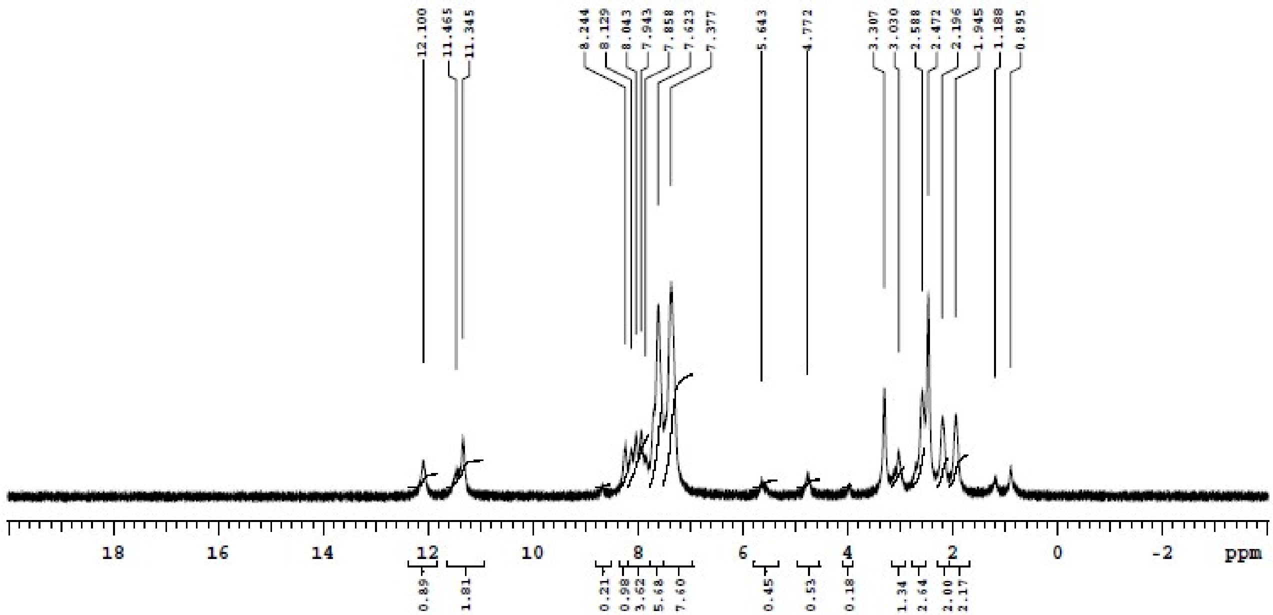

^{1}H NMR spectrum of compound 10

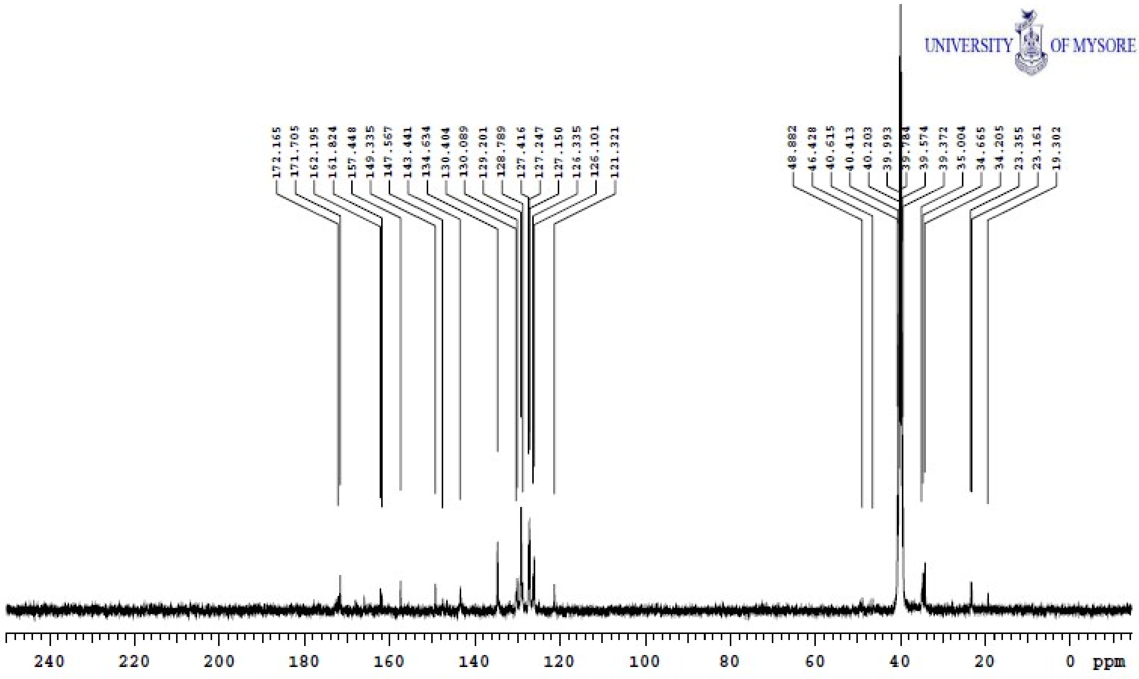

^{13}C NMR spectrum of compound 10

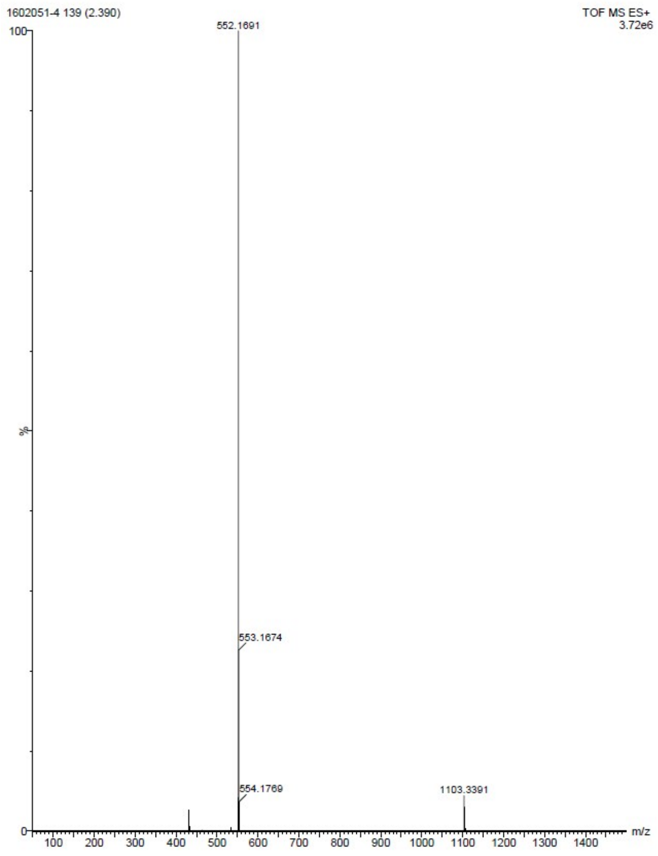

Mass spectrum of compound 10

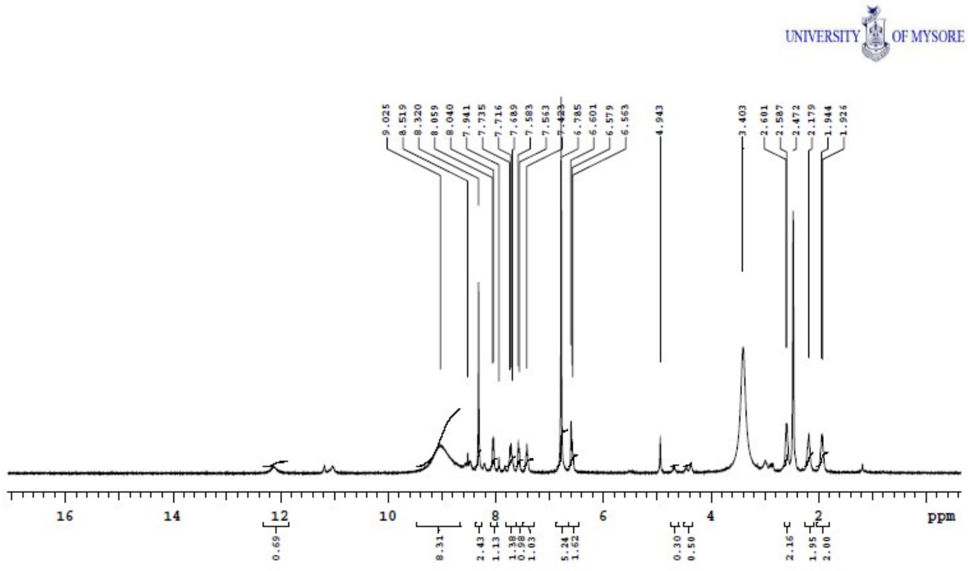

^{1}H NMR spectrum of compound 12

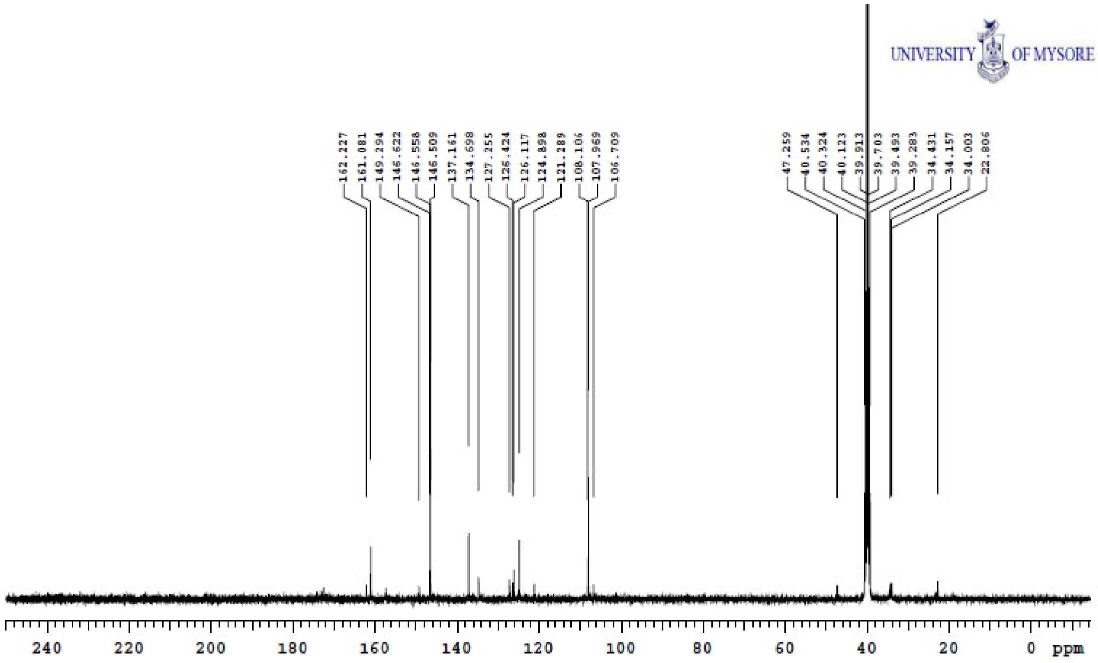

^{13}C NMR spectrum of compound 12

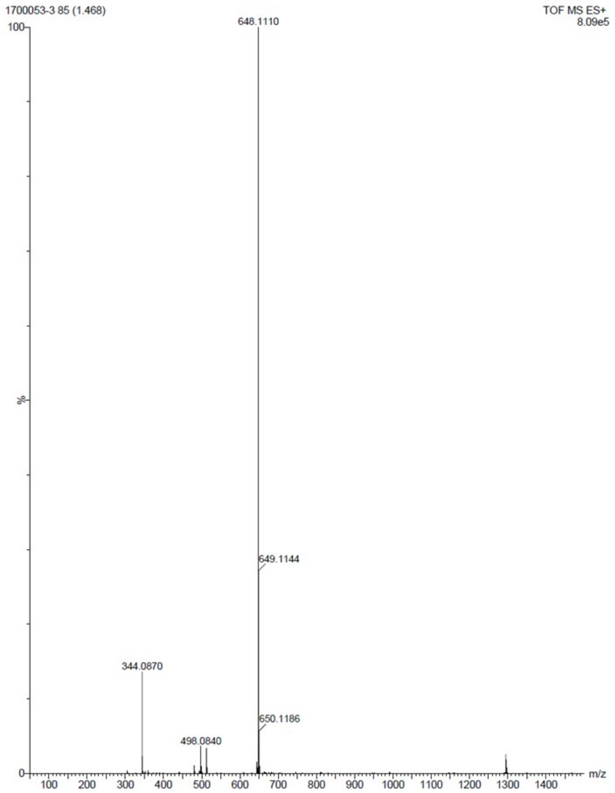

Mass spectrum of compound 12

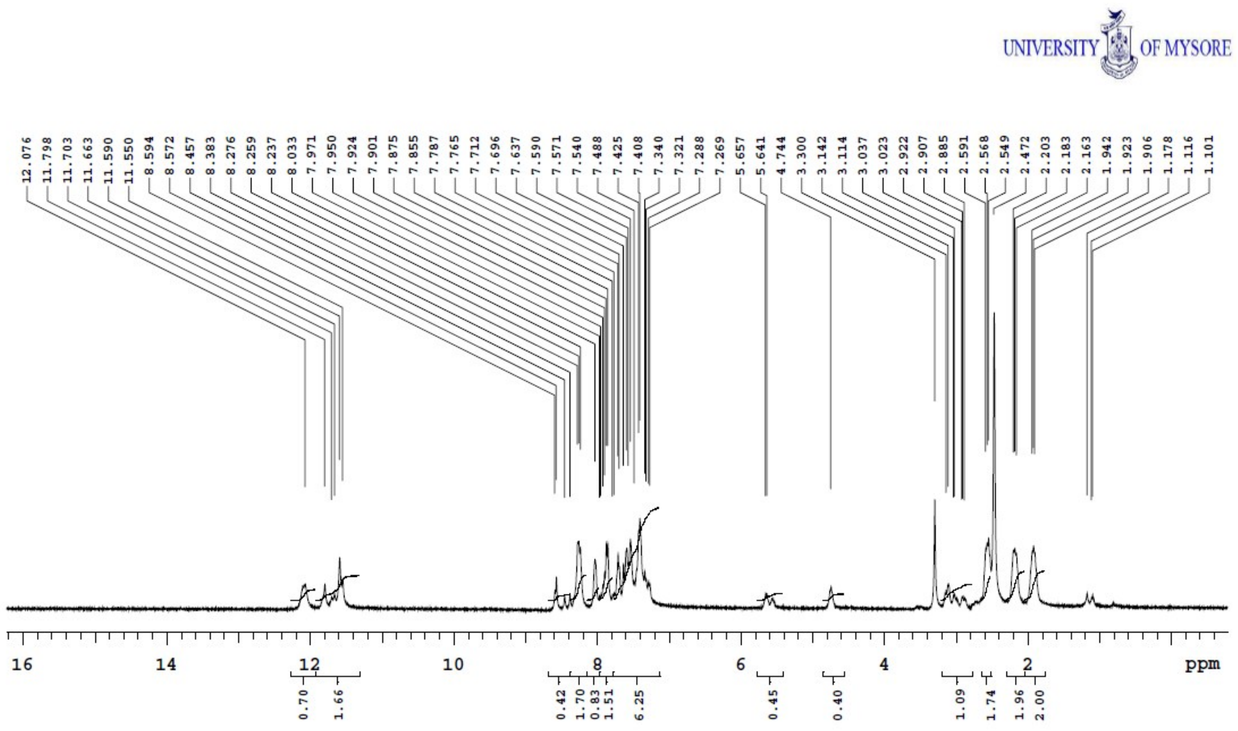

^{1}H NMR spectrum of compound 13

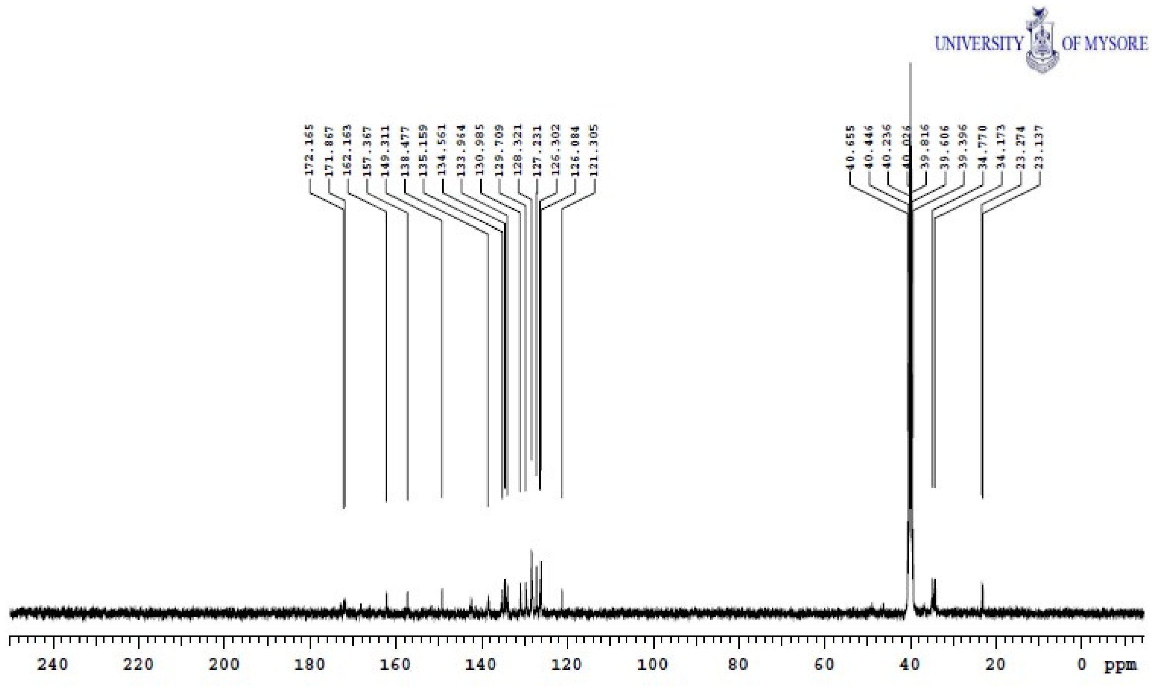

^{13}C NMR spectrum of compound 13

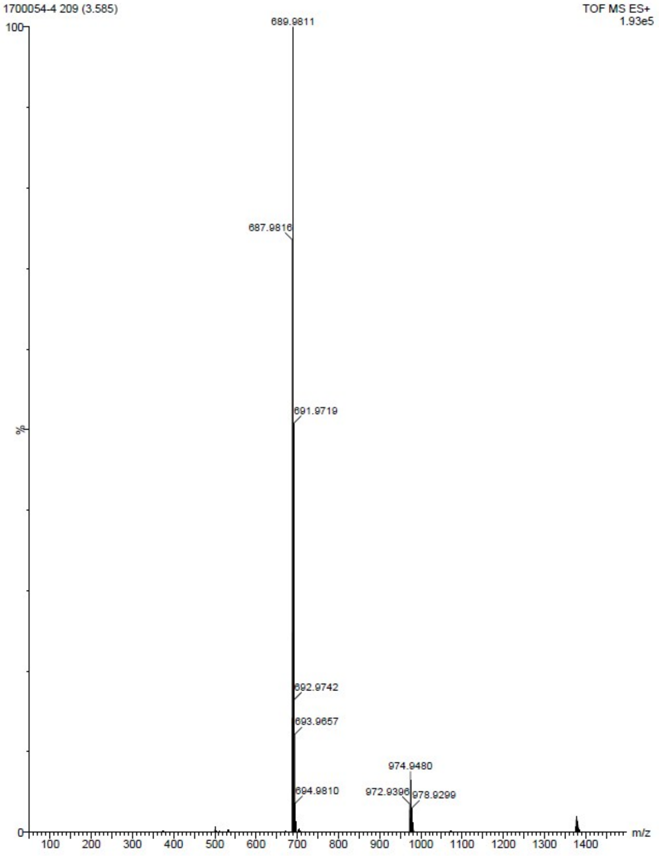

Mass spectrum of compound 13

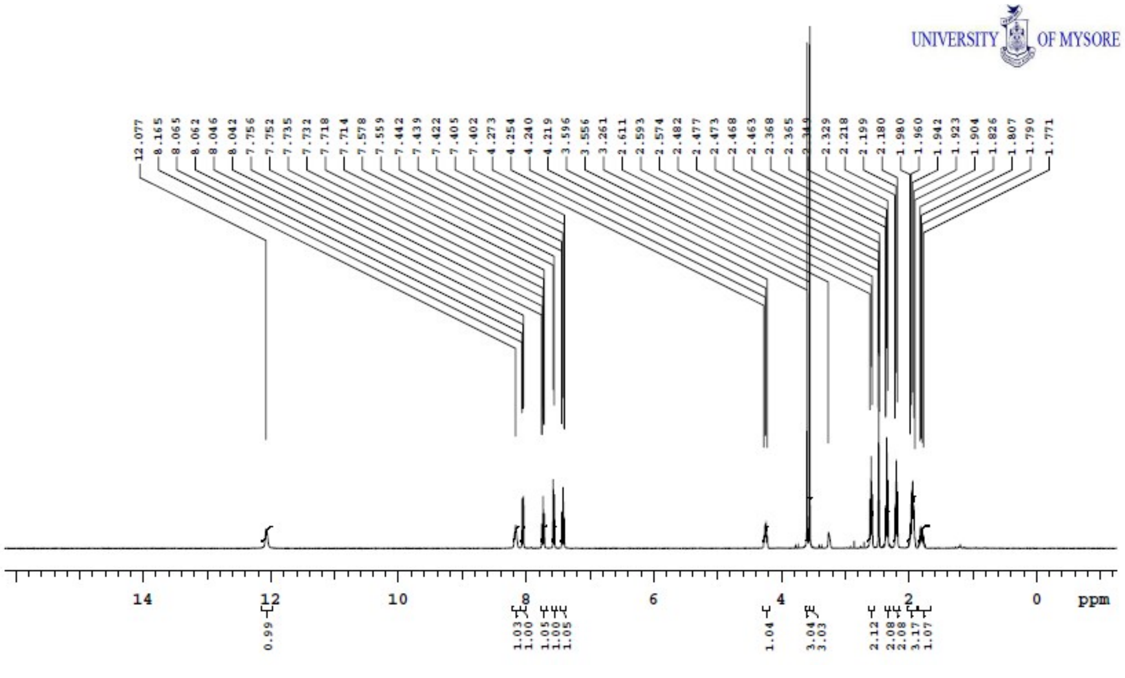

^{1}H NMR spectrum of compound 16

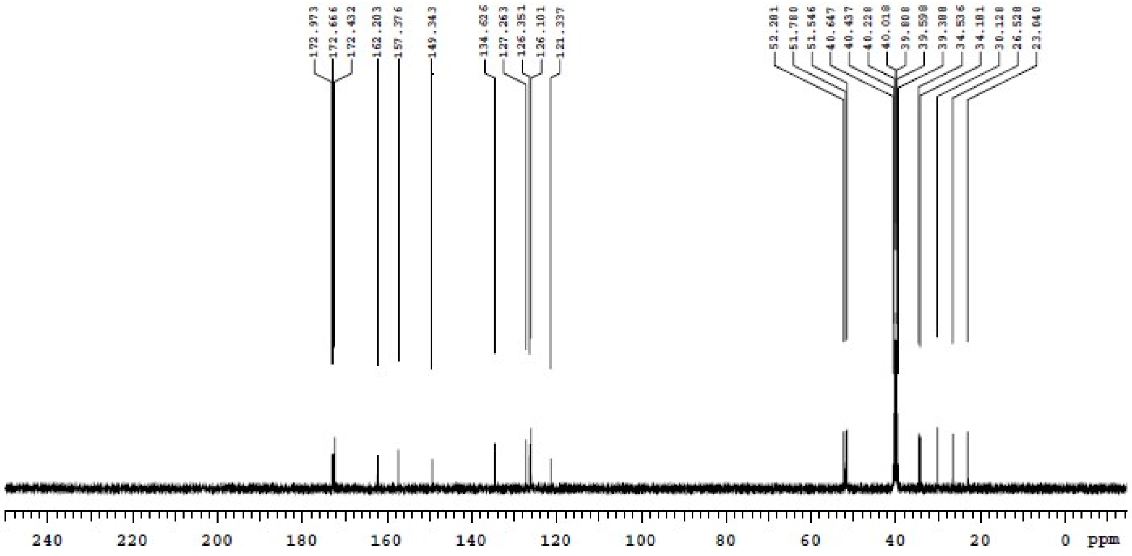

^{13}C NMR spectrum of compound 16

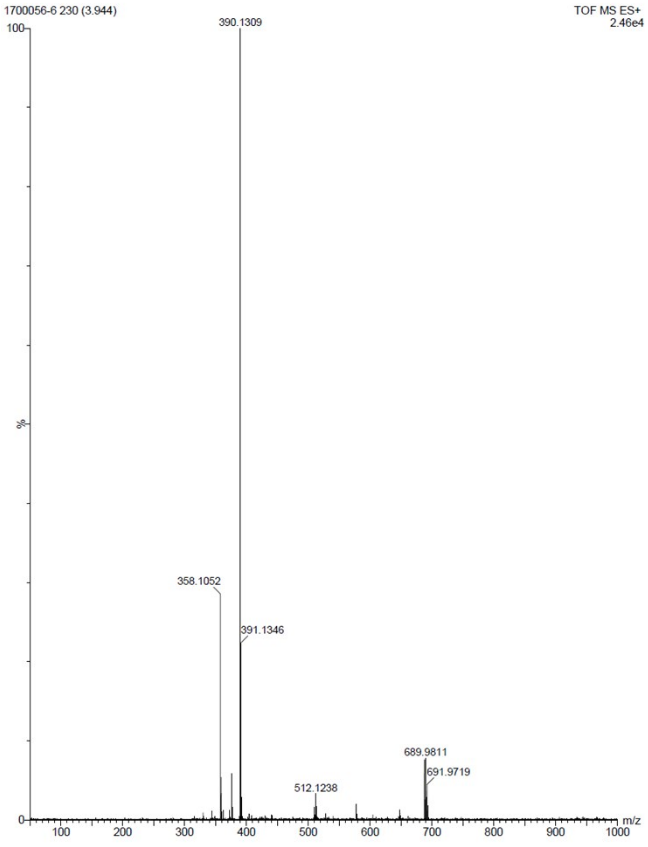

Mass spectrum of compound 16

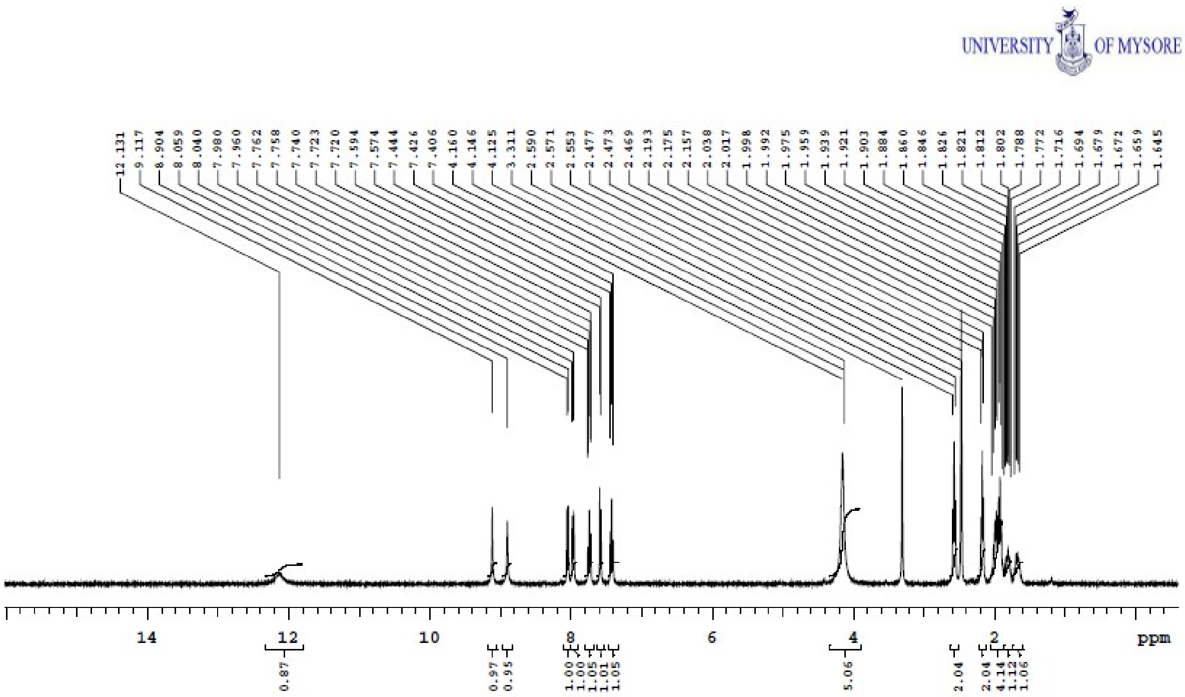

^{1}H NMR spectrum of compound 18

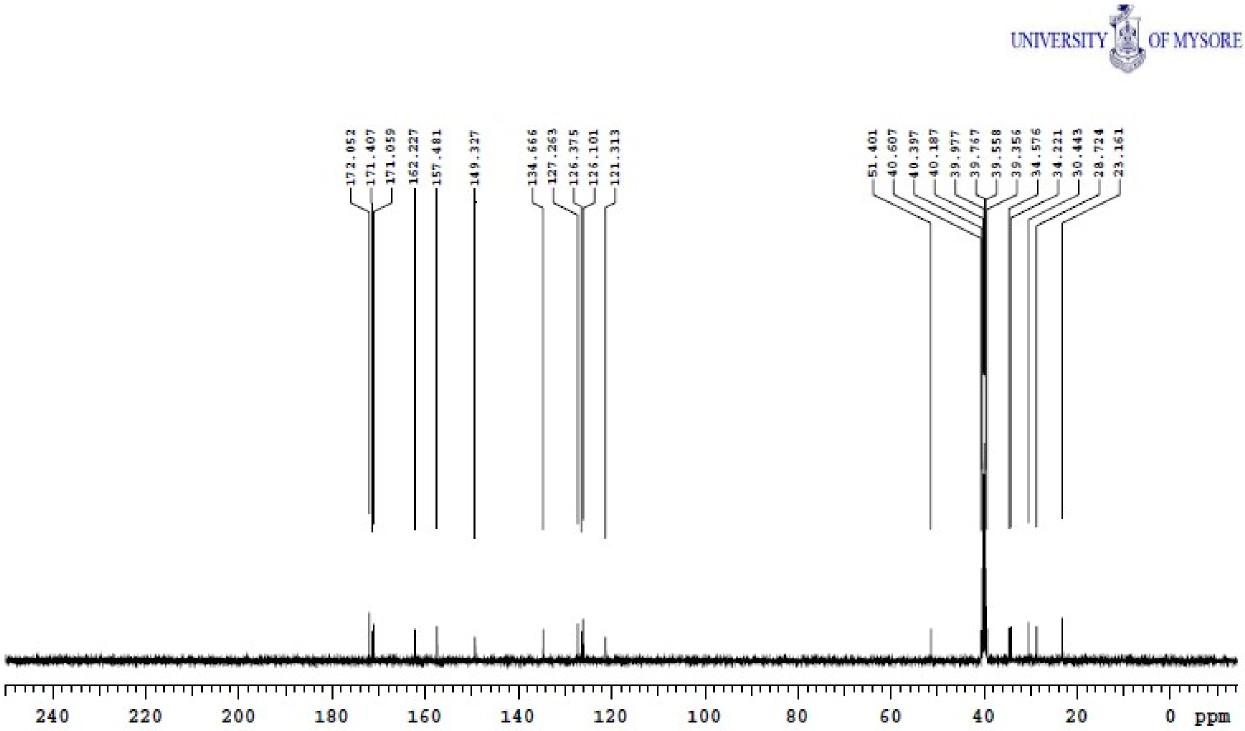

^{13}C NMR spectrum of compound 18

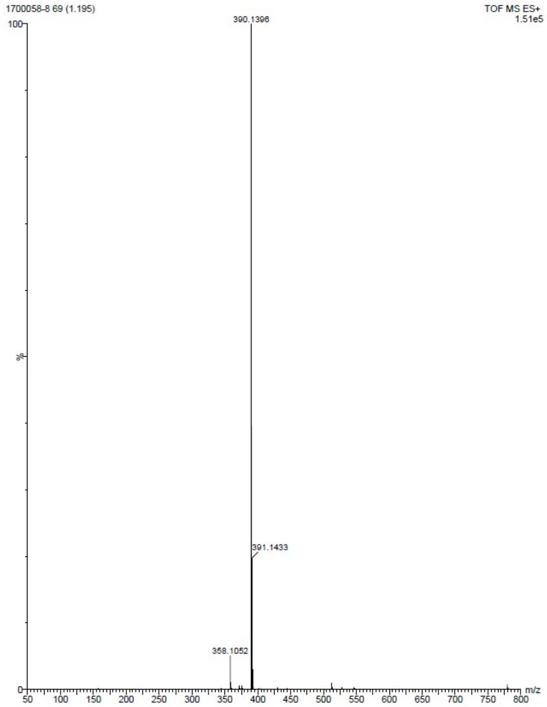

Mass spectrum of compound 18

UNIVERSITY OF MYSORE

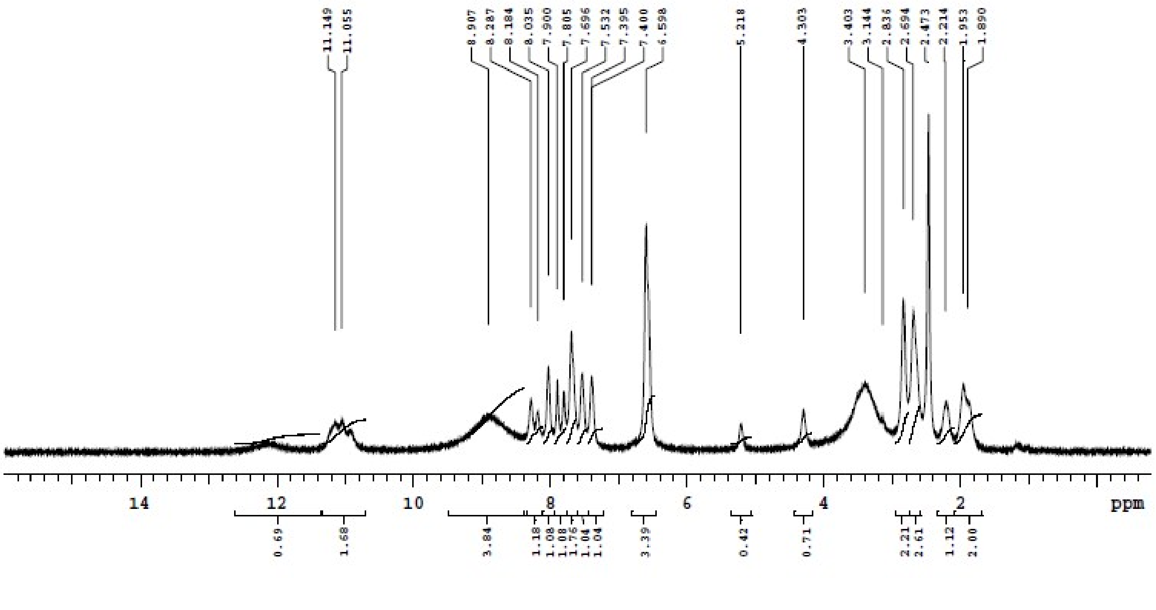

^{1}H NMR spectrum of compound 21

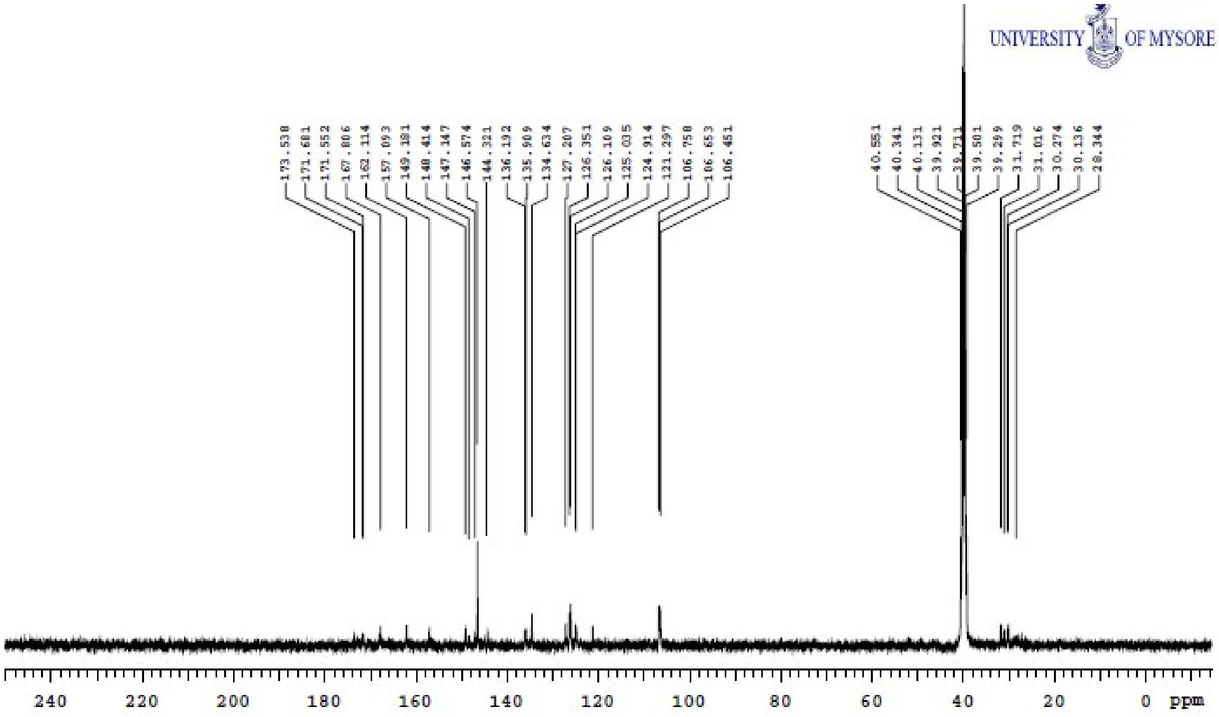

^{13}C NMR spectrum of compound 21

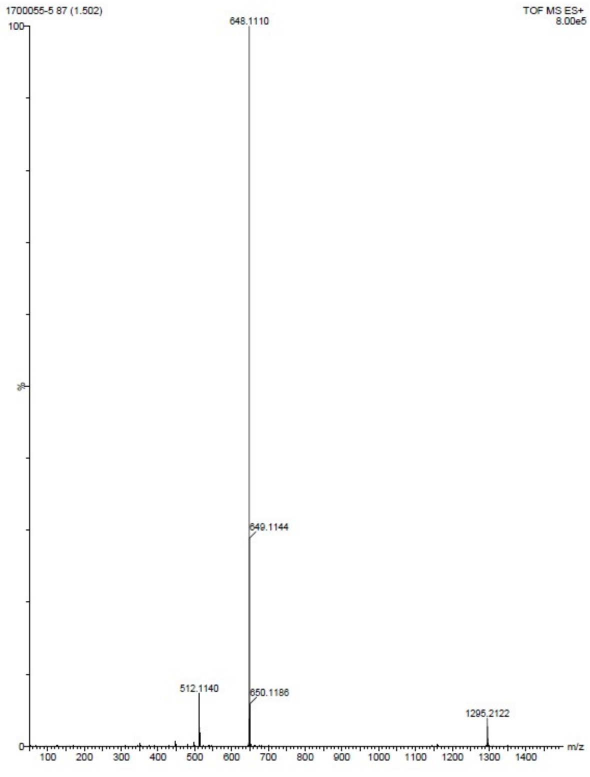

Mass spectrum of compound 21

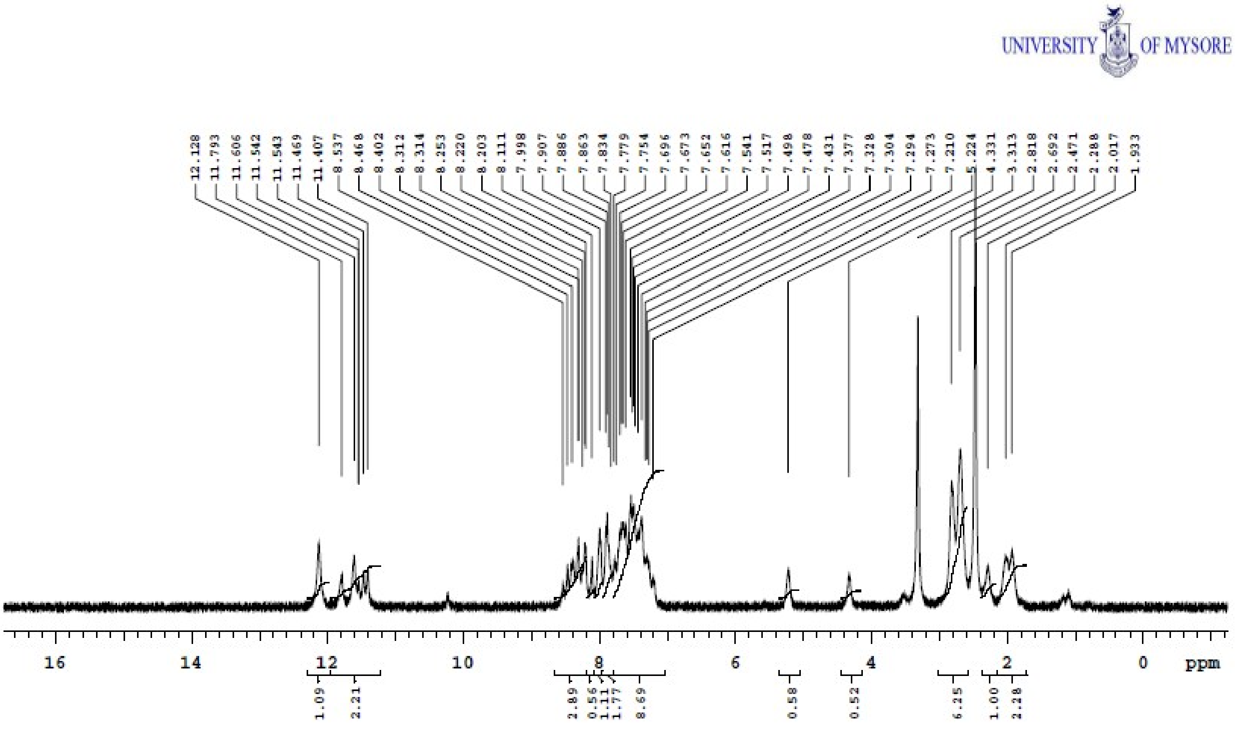

^{1}H NMR spectrum of compound 22

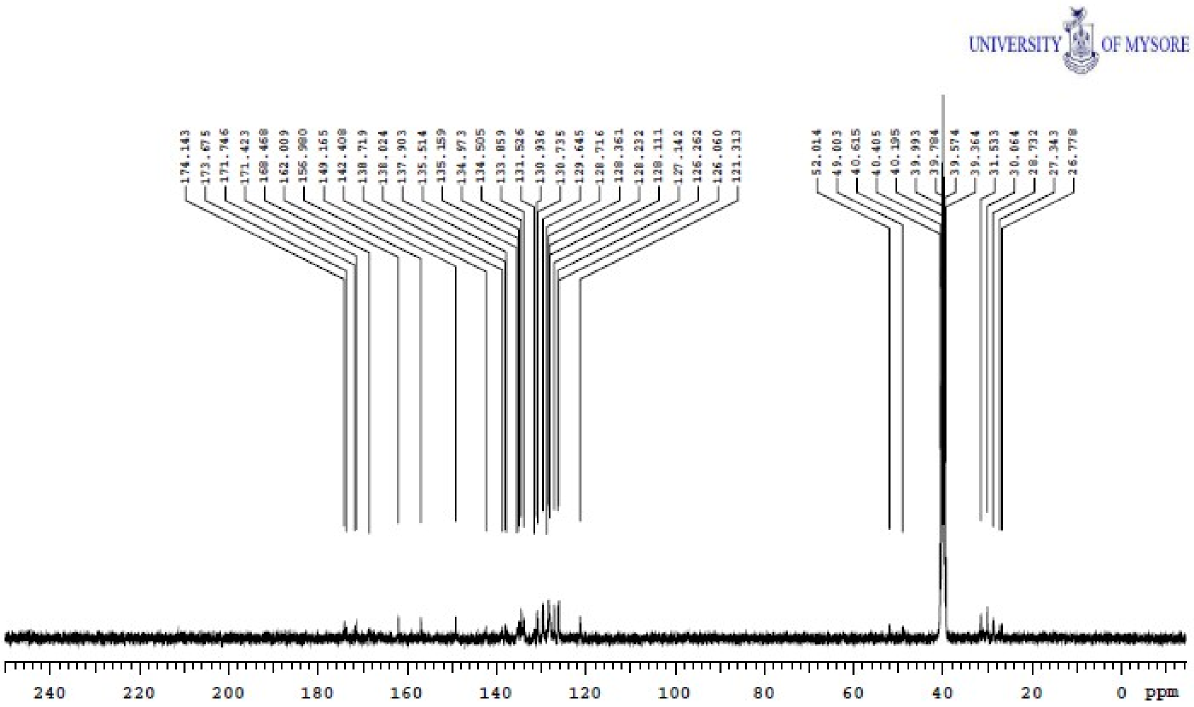

^{13}C NMR spectrum of compound 22

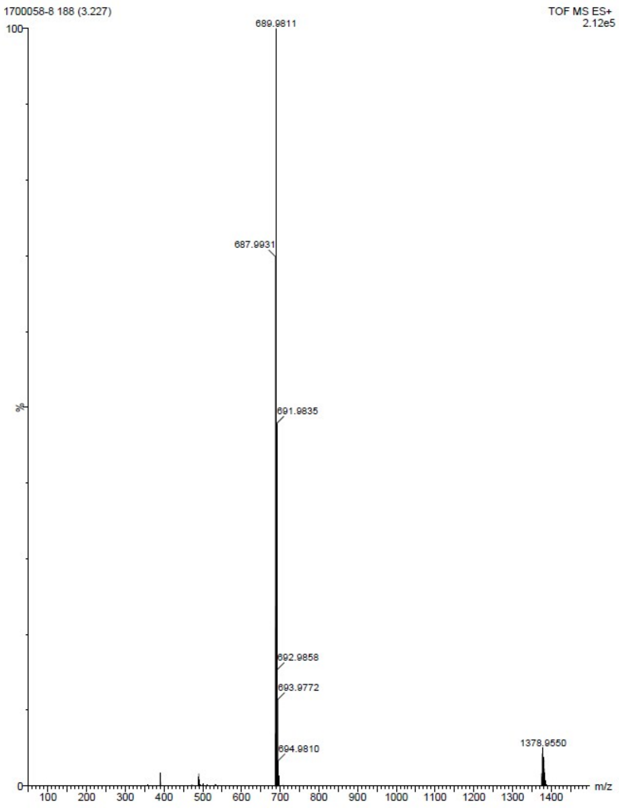

Mass spectrum of compound 22

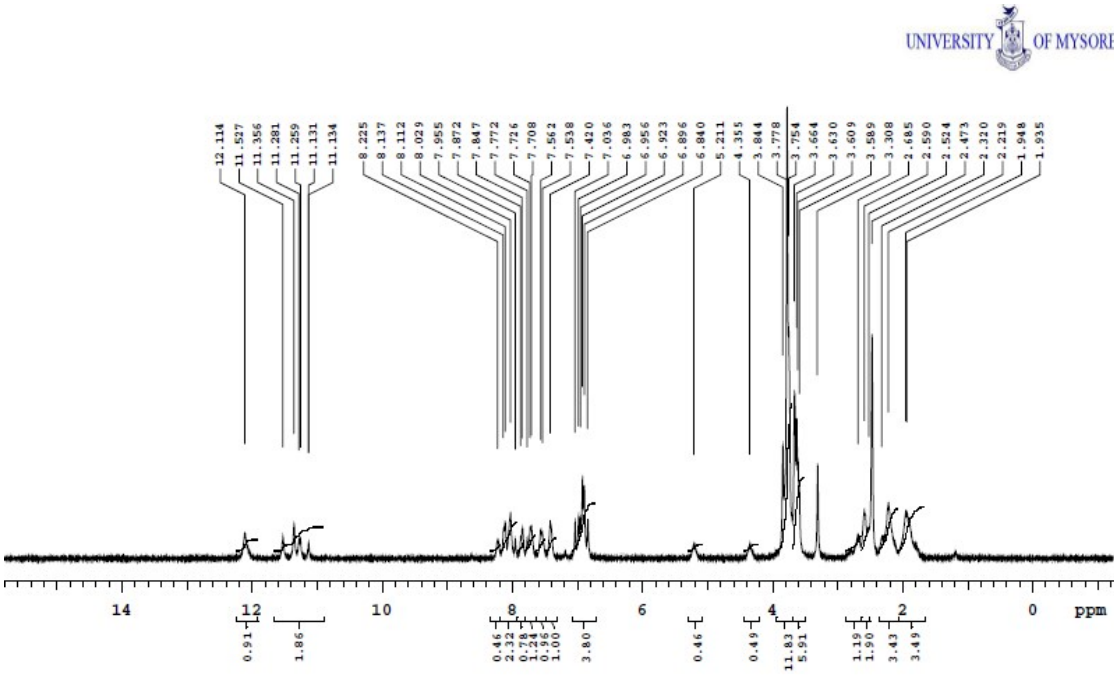

^{1}H NMR spectrum of compound 25

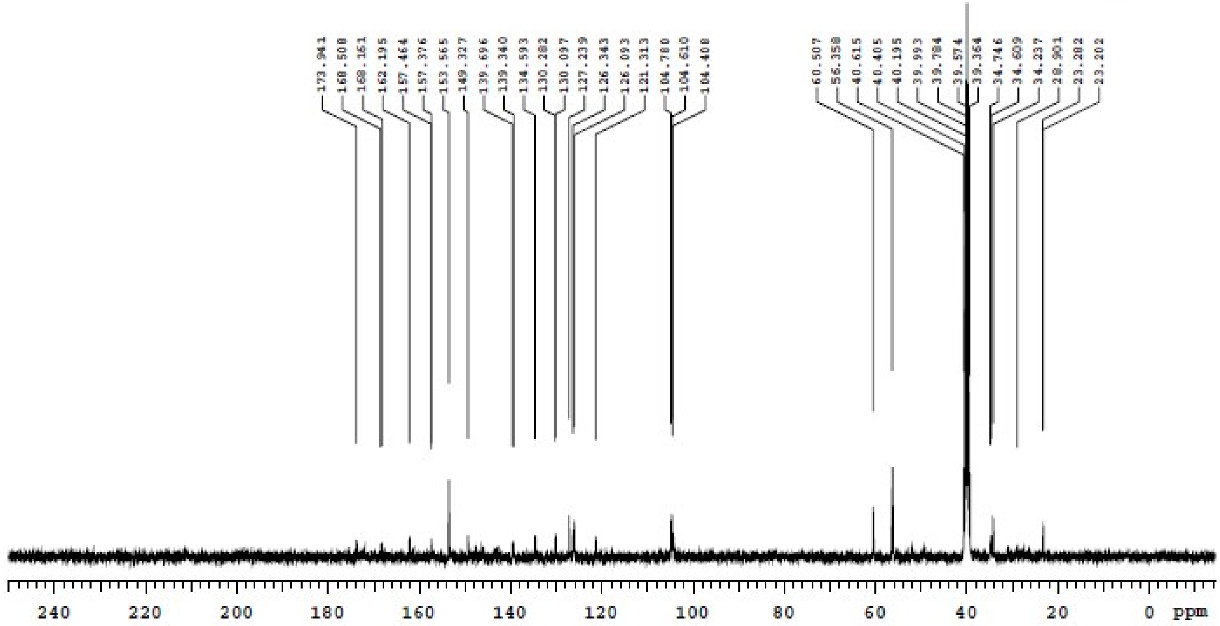

^{13}C NMR spectrum of compound 25

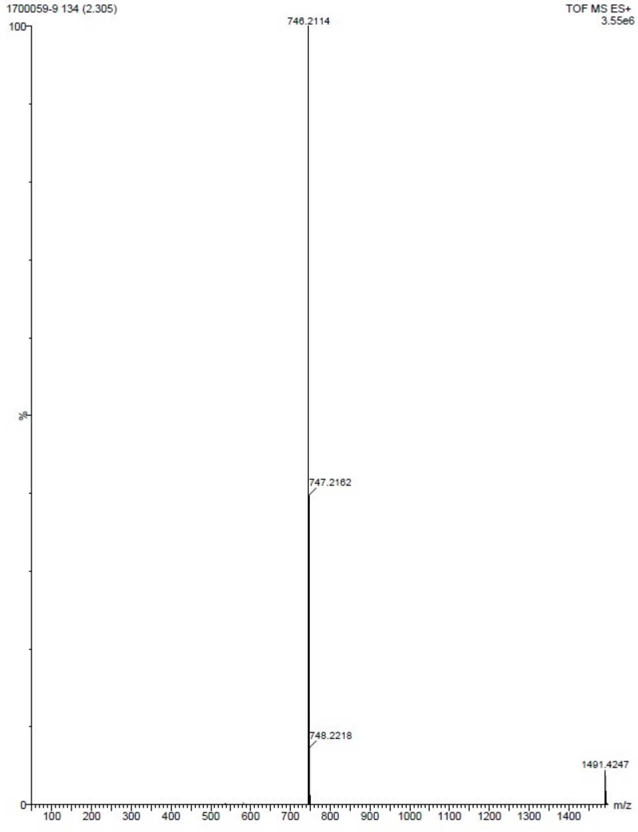

Mass spectrum of compound 25

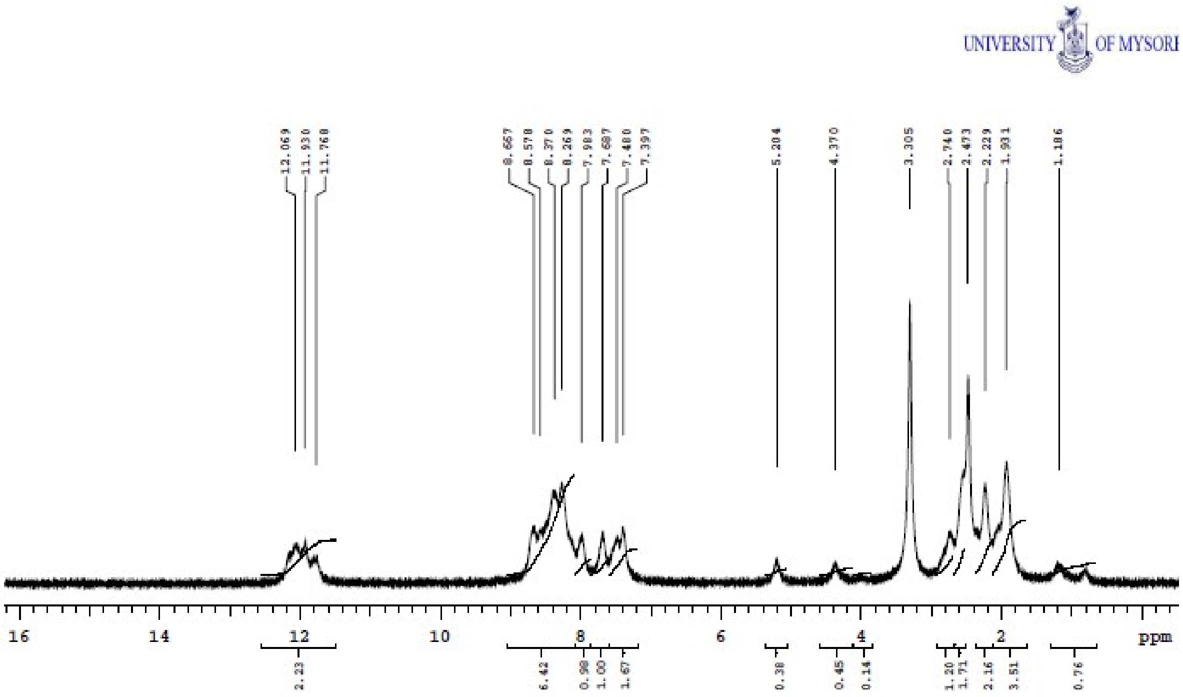

^1H NMR spectrum of compound 28

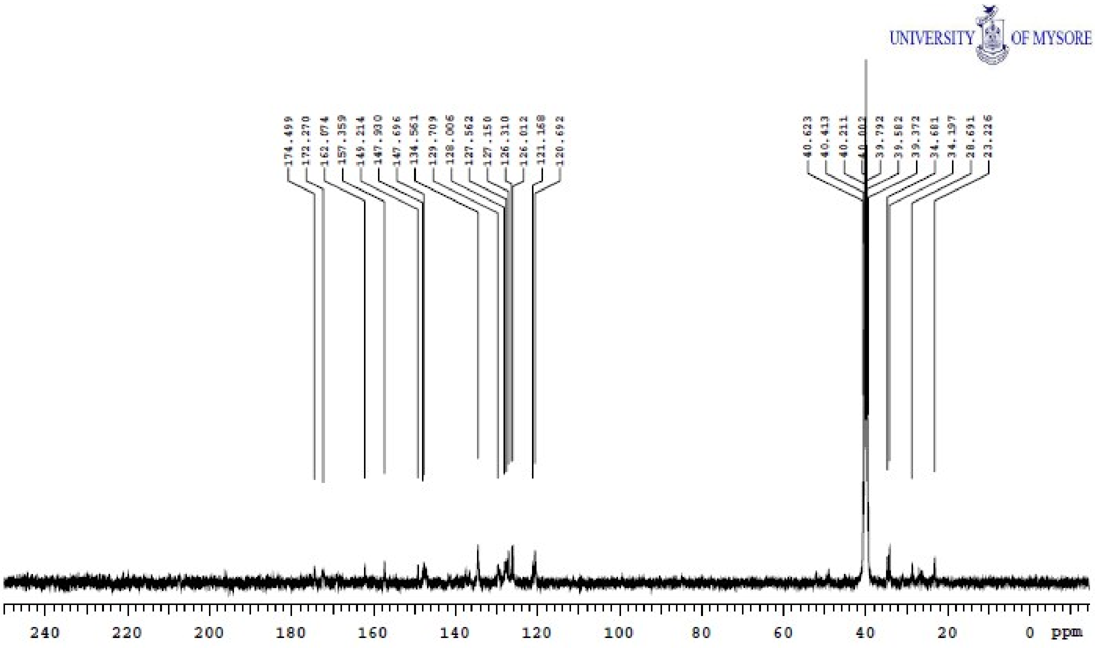

^{13}C NMR spectrum of compound 28

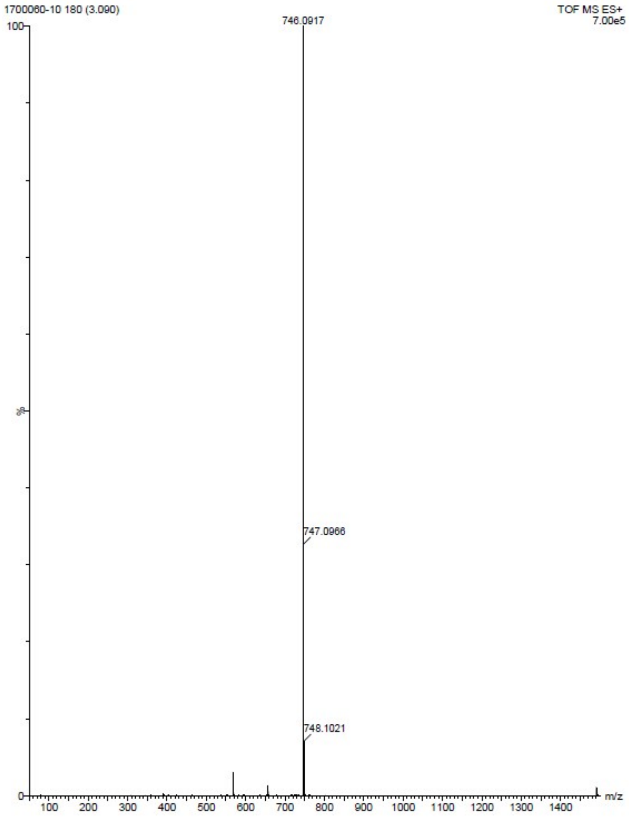

Mass spectrum of compound 28
